# Histamine deficiency delays ischaemic skeletal muscle regeneration via inducing aberrant inflammatory responses and repressing myoblast proliferation

**DOI:** 10.1111/jcmm.14720

**Published:** 2019-10-10

**Authors:** Mieradilijiang Abudupataer, Weihong Zou, Weiwei Zhang, Suling Ding, Zheliang Zhou, Jinmiao Chen, Hui Li, Zhiwei Zhang, Chunsheng Wang, Junbo Ge, Tao Hong, Xiangdong Yang

**Affiliations:** ^1^ Shanghai Institute of Cardiovascular Diseases Zhongshan Hospital Fudan University Shanghai China; ^2^ Department of Cardiac Surgery Zhongshan Hospital Fudan University Shanghai China; ^3^ Department of Pharmacy The First Affiliated Hospital University of South China Hengyang China; ^4^ Institutes of Biomedical Sciences Fudan University Shanghai China

**Keywords:** C2C12 myoblast cell, histamine, IGF‐1, regeneration, satellite cell, skeletal muscle

## Abstract

Histidine decarboxylase (HDC) catalyses the formation of histamine from L‐histidine. Histamine is a biogenic amine involved in many physiological and pathological processes, but its role in the regeneration of skeletal muscles has not been thoroughly clarified. Here, using a murine model of hindlimb ischaemia, we show that histamine deficiency in *Hdc* knockout (*Hdc^−/−^*) mice significantly reduces blood perfusion and impairs muscle regeneration. Using *Hdc*‐EGFP transgenic mice, we demonstrate that HDC is expressed predominately in CD11b^+^Gr‐1^+^ myeloid cells but not in skeletal muscles and endothelial cells. Large amounts of HDC‐expressing CD11b^+^ myeloid cells are rapidly recruited to injured and inflamed muscles. *Hdc^−/−^* enhances inflammatory responses and inhibits macrophage differentiation. Mechanically, we demonstrate that histamine deficiency decreases IGF‐1 (insulin‐like growth factor 1) levels and diminishes myoblast proliferation via H3R/PI3K/AKT‐dependent signalling. These results indicate a novel role for HDC‐expressing CD11b^+^ myeloid cells and histamine in myoblast proliferation and skeletal muscle regeneration.

## 1 INTRODUCTION

Skeletal muscle regeneration after ischaemic injury involves multiple processes, specifically the inflammatory response, angiogenesis and tissue regeneration. Skeletal muscles have a robust capacity for regeneration, so they can repair themselves by regenerating new muscle fibres after severe damage. It has been documented that skeletal muscle regeneration ability relies on the proliferation of tissue‐resident stem cells (satellite cells) and a suitable stem cell survival environment.^1^ Satellite cells normally lie in a quiescent state and become activated upon muscle fibre damage. Satellite cells are self‐renewing to replenish the stem cell pool during regeneration.^2^ Following muscle injury, quiescent satellite cells are activated and differentiate to myoblasts, which enter the cell cycle and begin to proliferate and differentiate, eventually fusing to damaged myofibres and regenerating new muscle fibres.^3^ The growth factors and cytokines released from the injured blood vessels, muscle fibres and infiltrated immune cells have multiple effects on the proliferation and differentiation of myoblasts in response to muscle fibre damage.

A large amount of evidence has demonstrated that inflammation and the immune response play critical roles in muscle regenerative processes, and angiogenesis as well as appropriate extracellular matrix (ECM) are both essential for co‐ordinate tissue regeneration.^4^, ^5^ At the early stage of tissue damage, neutrophils and macrophages are the predominant immune cells recruited to the injured site and help to clean the necrotic tissue debris. Following the onset of neutrophil infiltration, M1‐type macrophages are recruited to the injured site and phagocytize the damaged muscle fibres. Macrophages also secrete cytokines such as TNF‐α, which initiates muscle regeneration and the production of growth factors such as IGF‐1 and PDGF, to promote myoblast proliferation.^6^, ^7^, ^8^ The literature has reported that macrophage deletion or chemokine/chemokine receptor deficiency leads to an aberrant immune response and ultimately hampers the regeneration of muscle fibre.^5^, ^9^, ^10^ Therefore, innate immune cells seem to play an important role in regulating muscle regeneration by producing cytokines or directly activating tissue stem cells.

Apart from inflammation, essential growth factors such as IGF‐1 are indispensable for muscle growth and regeneration. As a potent mitogen, IGF‐1 can promote both the proliferation and differentiation of myoblasts in vivo and in vitro by binding to the type I IGF receptor (IGF‐IR).^11^, ^12^ The activated IGF‐IR stimulates the adaptor molecule IRS‐1 to further activate multiple intracellular signal transduction cascades, including the phosphatidylinositol 3‐kinase (PI3K)‐AKT cascade and the Raf‐Mek‐Erk1/2 cascade.^13^ Infiltration of inflammatory cells, which secrete cytokines and growth factors, to the injury site influences local blood flow, the inflammatory response and cell growth at that site. Likewise, muscle cells can also release some inflammatory factors, which influence the inflammatory response by playing an active role in modulating inflammatory processes. For instance, recent evidence showed that interleukin‐4 (IL‐4), whose expression is dependent on NFAT signalling, acts as a myoblast recruitment factor during muscle growth, and IGF‐1 has also been shown to activate NFAT.^14^ Therefore, IGF‐1 can promote the production of specific cytokines in muscle. Injured muscle fibres can increase the infiltration of immune cells in injured muscle, and myoblasts have been demonstrated to release factors that attract macrophages and monocytes. This indicates that myoblasts, immune cells and IGF‐1 can interact with each other and influence their function, resulting in the formation of a microenvironment that regulates muscle regeneration co‐ordinately.^15^, ^16^

Histamine has been documented to play critical roles in many biological and pathological processes; it is synthesized from the amino acid L‐histidine by the action of a unique enzyme, histidine decarboxylase (HDC).^17^, ^18^ The gastric enterochromaffin‐like cells, histaminergic neurons, mast cells and basophiles are important cellular sources of histamine. Interestingly, recent studies reported that HDC is highly expressed in CD11b^+^Gr‐1^+^ immature myeloid cells within the bone marrow and spleen, and these HDC‐expressing CD11b^+^ immune cells can be activated and recruited to inflammatory tissues.^19^, ^20^ Histamine is involved in the regulation of inflammation, hematopoiesis, immune cell differentiation, embryonic development and wound healing, but its roles in the proliferation and differentiation of muscle stem cells and tissue regeneration have not been fully studied. A recent study found that histamine therapy reduces muscle injury and improves grip strength of *Dmd^mdx^* mice, but its underlying mechanism has not been reported.^21^ In the early‐phase of myoblast differentiation in vitro, high levels of histamine receptor expression can be detected, suggesting that histamine may participate in myoblast proliferation.^22^ A previous study also reported that histamine could stimulate the expression of IGF‐1 in human glioma cells.^23^ These studies suggest that histamine may have an impact on the proliferation and differentiation of myoblasts and muscle regeneration. However, to clarify the roles of HDC^+^CD11b^+^ immune cells and histamine in the proliferation and differentiation of myoblasts and muscle regeneration, further investigation is needed.

In the current study, *Hdc*‐EGFP transgenic mice and histamine‐deficient *Hdc* knockout mice were applied to investigate the expression and function of HDC^+^ cells and histamine in the wound healing response to a model of mouse hindlimb ischaemia. Here, we report that histamine deficiency promotes inflammation, reduces limb perfusion and represses myoblast proliferation, leading to delayed muscle regeneration after ischaemic injury of skeletal muscle.

## 2 MATERIALS AND METHODS

### 2.1 Animals

*Hdc*‐EGFP, *Hdc*‐CreERTM: Rosa26mTmGFP and HDC knockout (*Hdc^−/−^*) mice were generously provided by Professor Timothy C. Wang from Columbia University. The generation of *Hdc*‐EGFP and *Hdc^−/−^* mice has been described in previous papers.^19^, ^24^ Balb/C mice and C57BL/6 mice were purchased from the Department of Laboratory Animal Science, Fudan University, to serve as background controls. The background of Hdc*^−/−^* mice is Balb/C and that of Hdc‐EGFP mice is C57BL/6; these were imported from our collaborator at Columbia University. In the present study, we chose the appropriate control mice for different mouse models. All mice were housed under specific pathogen‐free conditions in an animal room with a 12/12‐hours day/night cycle with free access to water and food. This study was performed in strict accordance with the recommendations from the Guide for Animal Management Rules from the Ministry of Health of the People's Republic of China. The protocol was approved by the Committee on the Ethics of Animal Experiments of Fudan University (approval reference number: SY2014.2.001.002).

### 2.2 Hindlimb Ischaemia model

Animals, aged 8‐10 weeks, were anaesthetized by subcutaneous injection of 1% pentobarbital sodium. Then, each animal was placed in the supine position on the pre‐operating table. Hair removal cream was applied to thoroughly remove the hair from the hindlimb. Using aseptic technique, the femoral artery was found, and the neurovascular bundle was exposed. Then, the femoral artery was separated from the femoral vein and nerve. The right femoral artery was ligated proximal to the inguinal ligament and distal to the popliteal artery by a strand of 6‐0 silk suture. Superficial branches and bifurcation of the femoral artery were cauterized, and the segment of femoral artery between the distal and proximal knots was transected. After the incision was closed, the animal was placed on a heated pad and monitored continuously until awake. At D1 post‐surgery when the animal had recovered, a laser Doppler blood perfusion process was performed to confirm the ischaemia of the limb.^25^

### 2.3 Laser doppler perfusion imaging

Non‐invasive measurements of hindlimb perfusion were scanned before and 1, 7, 14, and 21 days after ligation using a laser Doppler perfusion imager (PeriScan PIM 3 system, Perimed). Animals were anaesthetized by 1% pentobarbital sodium injected subcutaneously and were placed on an isothermal heating pad. The results were expressed as a ratio of perfusion in the right (ischaemic limb [IL]) versus left (non‐ischaemic limb [NIL]) limb to avoid the influence of light and temperature. At each time‐point, each animal was scanned three times to obtain the average ratio.^26^

### 2.4 Assessment of limb ischaemic damage

Semi‐quantitative measurement of ischaemic damage (necrosis score) was also performed using the following criteria: 0 = no necrosis; 1 = nail necrosis; 2 = toe necrosis/amputation; 3 = forefoot necrosis/amputation; and 4 = above ankle necrosis/amputation.

### 2.5 Exogenous histamine injection and tamoxifen injection

Our previous study confirmed that intraperitoneal injection of histamine at the dose of 4 mg/kg every 2 days could increase the levels of histamine in the peripheral blood of Hdc*^−/−^* mice, approaching the levels in WT mice (Deng et al, 2015). Hdc*^−/−^* mice were treated with 4 mg/kg histamine (Sigma) for three days before limb ischaemia and 4 mg/kg histamine every 2 days after limb ischaemia. Hdc‐CreERTM:Rosa26mTmGFP mice were injected subcutaneously with 4 mg/kg tamoxifen (Sigma), diluted in corn oil, per day for 3 consecutive days before limb ischaemia. WT mice were treated with 1 mg/kg Histamine H3 receptor antagonist (ciproxifan, H3A) for 3 days before limb ischaemia and every 2 days after limb ischaemia.

### 2.6 Flow cytometry

The blood, spleen and bone marrow were taken from hindlimb ischaemic *Hdc^−/−^* and WT mice at D3 and D7 after the implementation of the animal model. Red blood cells in peripheral blood were fragmented by erythrocyte lysis, and other nucleated cells were collected. The spleen was ground and filtered through 40‐μm strainers. Bone marrow was flushed with ice‐cold phosphate buffer saline (PBS, Sangon Biotech) and filtrated through 40‐μm strainers. Using RBC lysis buffer (BD Biosciences), red blood cells were destroyed among the cells collected from spleen and bone marrow. Cells were stained with a mixture of antibodies (anti‐CD11b‐APC, anti‐Gr‐1‐PerCP‐Cy5.5, anti‐Ly6C‐PE and anti‐F4/80‐FITC BD Biosciences), and data were acquired using an LSRII flow cytometer (BD Biosciences) and were analysed with FlowJo7 software (Tree Star Inc).

### 2.7 Centrally located myocyte nuclei

Haematoxylin and eosin (H&E) staining of gastrocnemius muscle regeneration was confirmed by the presence of multiple, centrally located myocyte nuclei. The number of centrally nucleated myofibres was counted according to the following standard: ten fields for per gastrocnemius muscle were randomly selected, the number of centrally nucleated myofibres was counted in each field, and the average number of ten fields was taken. The observers were blinded.

### 2.8 Immunofluorescence and immunohistochemistry assay

Gastrocnemius muscles from *Hdc*‐EGFP and *Hdc*‐CreERTM:Rosa26mTmGFP mice were fixed with 4% paraformaldehyde for 6‐12 hours and incubated overnight in 30% sucrose to prepare frozen tissue. Primary antibodies to CD11b (Abcam) and PAX‐7 (Abcam) were used for immunofluorescence staining. *Hdc^−/−^* and WT mouse skeletal muscles were fixed with 10% formalin for paraffin embedding and with 4% paraformaldehyde for frozen tissue. H&E and Masson staining were performed. Primary antibodies to CD11b (Abcam) were used for immunohistochemistry to detect inflammatory cells. CD34 (Abcam) and Ki67 (Abcam) antibodies were used to identify proliferating cells. Primary antibodies to PAX‐7 (Abcam) and MyoD (Abcam) were used for immunofluorescence staining to detect myoblast proliferation. The number of cells was counted according to the following standard: five fields were randomly selected, the number of cells in each field was counted, and the average number of five fields was taken. The observers were blinded.

### 2.9 Quantitative real‐time PCR (qRT‐PCR)

Total RNA from quadriceps femoris and gastrocnemius muscles and C2C12 cells was extracted using TRIzol reagent (Invitrogen). RNA was converted into cDNA using an RT Kit (Takara). Gene expression of *IL‐1β*, *Il‐6*, *Tnf‐α*, *Il‐10*, *Tgf‐β*, *Igf‐1*, *Pi3k*, *Akt*, *Pkd1* and *P70s6k* was analysed using SYBR Premix Ex Taq (Takara) on an Applied Biosystems AB 7500 Real‐Time PCR System. The final results were calculated from an average of three independent values of each gene by standardizing to GAPDH control values. The PCR primers (Sangon Biotech) are listed in the Supplemental Information.

### 2.10 Western blot

Quadriceps femoris and gastrocnemius muscles of *Hdc^−/−^* and WT mice were cut into pieces first and then lysed with RIPA lysis buffer (Beyotime Biotechnology) for 30 minutes. C2C12 cells were directly lysed by RIPA for 30 minutes, and the subsequent procedure was the same as the tissue procedure. Lysates were then centrifuged at 14 000 *g* for 25 minutes. The protein concentration was calculated using a BCA protein assay (Well Biotech). Extracted proteins were diluted in sample loading buffer and heated for 5 minutes at 95℃. Equal masses of proteins were separated by running on a 6%‐15% SDS–polyacrylamide gel electrophoresis gel and transferred onto polyvinylidene difluoride membranes. Membranes were then probed with primary antibodies: glyceraldehyde‐3‐phosphate dehydrogenase (GAPDH) (ab8245, Abcam), PAX‐7 (ab187339, Abcam), MyoD (ab16148, Abcam), VEGF (ab1316, Abcam), IGF‐1R (Novus Biologicals, G22121), PI3K (ab189403, Abcam), AKT (ab8805, Abcam) and P‐AKT (Cell Signaling, 4060T). Then, the membranes were incubated with a 1:2000 dilution of horseradish peroxidase‐conjugated goat anti‐rabbit and goat antimouse IgG‐secondary antibodies (Biotech Well) for 1 hour at room temperature. Bands were detected by the Super Signal chemiluminescence reagent substrate (Millipore, 1614601), and quantitative estimation of the bands’ intensity was performed using ImageJ software.

### 2.11 Cell culture and CCK8 cell proliferation assay

The C2C12 cell line was provided by the Chinese Academy of Sciences in Shanghai. Cells were cultured in DMEM high‐glucose medium supplemented with 10% FCS. After treatment with histamine, the C2C12 cell proliferation rate was measured by CCK8 (Biotech Well) assay. One hundred microlitres of cell suspension was dispensed in a 96‐well plate, and each trial group had 3‐5 replicates. Different concentrations of histamine, IGF‐1 receptor antagonist (GSK1904529A), IRS antagonist (NT157), PI3K antagonist (LY294002) and histamine H1, H2, H3 receptor antagonist were added to the plate, which was incubated for 0.5, 1, 2 and 4 hours. Ten microlitres of CCK8 solution was added to each well and incubated for 1‐4 hours. The OD values were measured at 450 nm, and the results were repeated at least 3 times with the same experimental conditions.

### 2.12 Satellite cell isolation

The mice were euthanized following approved IACUC guidelines by giving an intraperitoneal injection of 2,2,2‐tribromoethanol at a dose of 250 mg/kg, followed by cervical dislocation. Hindlimb muscle was isolated under aseptic conditions. The muscles were minced and digested with digestion medium containing collagenase II in a 37°C shaker at 100 rpm for 1 hour. After digestion was complete, the tissue was centrifuged at 1400 *g* for 5 minutes at room temperature. The digested muscle was resuspended and pipetted 20‐30 times to ensure that most myoblasts were released from the muscle tissue. The cell mixture was passed through 70‐μm and 30‐μm strainers, and the cells were resuspended in medium containing MGM + bFGF. The cells were seeded onto a 10% Matrigel pre‐coated dish.

### 2.13 BrdU staining

The cultured C2C12 cells were placed at a density of 20 000 cells per well in a 24‐well plate. The plate was incubated until the cells grew to 50%‐60% of the well size. Different concentrations of histamine were added to the plate and incubated for 1‐4 hours. For each well, 10 μL of BrdU solution was added and incubated for 2‐4 hours. Culture medium was removed, and cells were fixed with 4% paraformaldehyde for 30 minutes. Anti‐BrdU‐PerCP‐Cy5.5 (BD Biosciences, 560809) antibody was used to label the proliferating cells, and cell nuclei were visualized by DAPI staining. Five fields were randomly selected, the number of cells in each field was counted, and the average number of five fields was taken. The observers were blinded.

### 2.14 Statistical analysis

At least three replicates for per phenotype animal were used per experiment, and the results are expressed as the mean ± SD. A minimum of three animals was used per group for protein, RNA and FACS analyses. Statistical analysis was performed with GraphPad Prism 6 software. Two‐tailed Student's *t* tests were used to compare values between two groups, and one‐way ANOVA followed by Tukey's post hoc test was used for comparison among more than two groups. Differences were considered significant at *P* < .05.

## 3 RESULTS

### 3.1 Histamine deficiency reduces blood perfusion and aggravates the injury of ischaemic limbs

**Figure 1:**
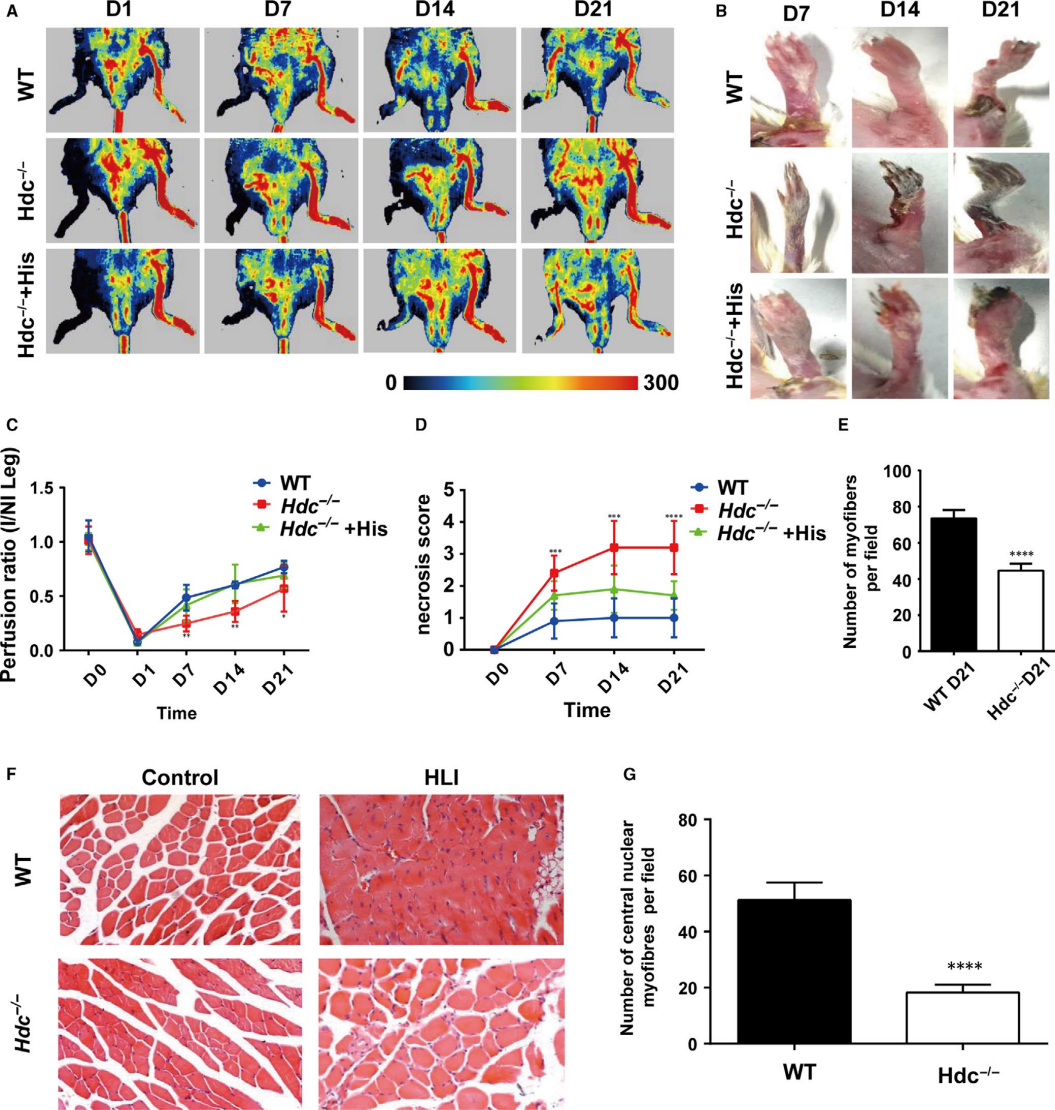
Histamine deficiency delays blood perfusion and functional recovery in ischaemic limbs. A, Laser Doppler scanning image at 1, 7, 14 and 21 days after femoral ligation. Red is the highest velocity, green is intermediate velocity, and blue is the lowest velocity. B, A picture of the ischaemic limb is shown at each point per group. Each photograph was taken under the same conditions. C, Ischaemic limb perfusion quantitation was analysed by the ratio of blood flow in the ischaemic limb to that in the non‐ischaemic limb at each time‐point. n = 8 mice per genotype. D, The necrosis score of the ischaemic limb was assessed by the range of necrosis. Scoring criteria are available in the Methods section. n = 8 mice per genotype. E, The number of fibres per muscle was counted at D21. F, Haematoxylin and eosin (H&E) staining of injured gastrocnemius muscle from *Hdc^−/−^*and WT mice at day 21 post‐injury. Scale bar, 50 µm. G, New regenerative myofibres were confirmed by the number of multiple, centrally located nuclear myocytes. Number of new fibres at day 21 per field of view. A significant reduction in the number of new fibres was observed in *Hdc^−/−^* mice relative to WT mice. n = 8 per genotype. For all experiments, error bars represent the mean ± SD.*P < .05, **P < .01, ***P < .001, ****P < .0001

To investigate the role of histamine in the repair of ischaemic limb injury, histamine‐deficient mice (*Hdc* knockout, *Hdc^−/−^*) and wild‐type mice (WT, Balb/c) were used to establish a muscle ischaemic model using standard vascular removal of the femoral artery. At baseline, the results for blood perfusion, capillaries and muscle fibres showed no significant differences in uninjured control limbs of WT and *Hdc^−/−^* mice. Consequently, blood flow was measured on day 1, day 7, day 14 and day 21 post‐operation by laser Doppler imaging. The results of the perfusion analysis revealed significantly delayed recovery of blood flow in ischaemic limbs of *Hdc^−/−^*mice relative to WT mice at 7, 14 and 21 days post‐injury (Figure 1A,C). Consistent with a low perfusion ratio, a high degree of limb necrosis was observed in *Hdc^−/−^* mice relative to WT mice (Figure 1B,D). To demonstrate the protective effect of histamine on the injured limb, exogenous histamine was administered in *Hdc^−/−^* mice before and after the surgical operation. As we expected, the administration of exogenous histamine in *Hdc^−/−^* mice significantly improved the ratio of blood perfusion (Figure 1A,C) and repressed the necrosis of the injured limb, almost reaching the level of the WT group (Figure 1B,D). The number of fibres per muscle was counted, and fewer fibres were examined in the muscle of *Hdc^‐/^^‐^* mice at D21 (Figure 1E). Furthermore, the results of haematoxylin and eosin (H&E) staining showed that most of the new skeletal muscles of WT mice had centrally located nuclei on day 21 post‐surgery, whereas few skeletal muscles had centrally located nuclei in *Hdc^−/−^*mice (Figure 1F,G). In addition, Masson's staining demonstrated more serious fibrosis in the injured limbs of *Hdc^−/−^*mice than WT mice (Figure S1). Taken together, these data suggest that HDC and histamine play critical roles in the injury and regeneration of ischaemic limb.

### 3.2 HDC is mainly expressed in CD11b^+^ myeloid cells rather than in muscle cells

**Figure 2:**
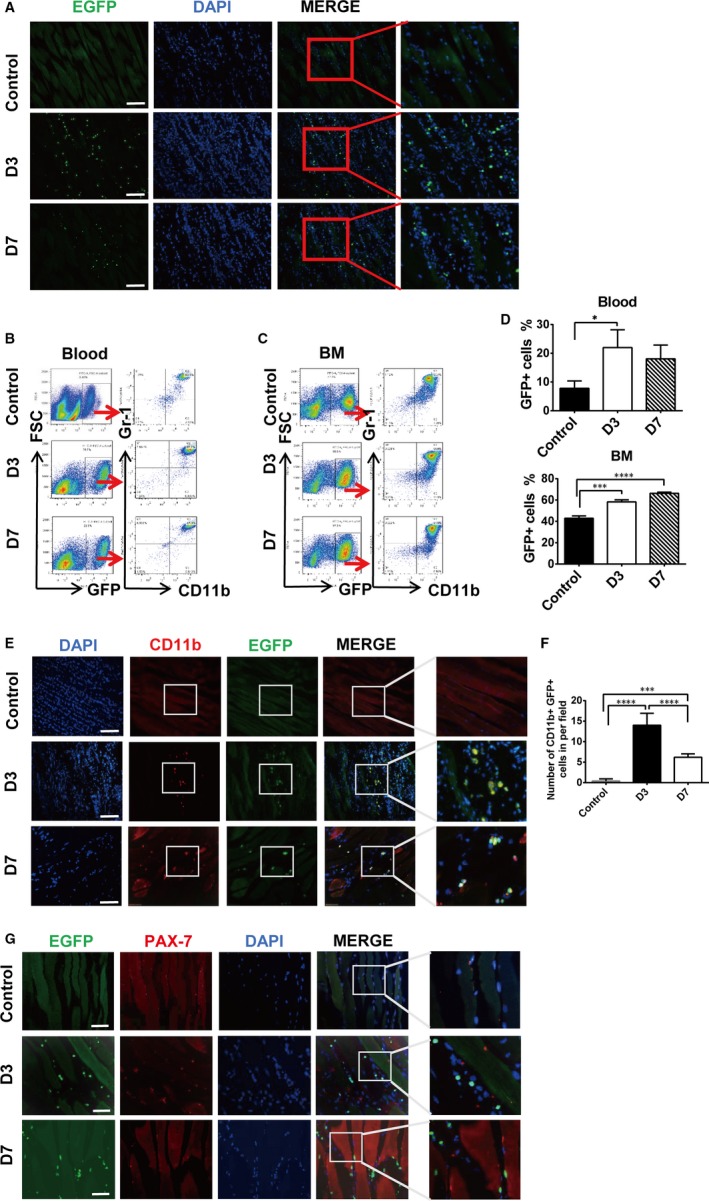
The expression of HDC in the injured limb. A, Immunofluorescence sections from ischaemic gastrocnemius muscle of *Hdc*‐EGFP mice at D3 and D7 post‐ischaemia and control non‐ischaemic muscle of *Hdc*‐EGFP mice. Top: EGFP^+^ cells were not observed in the muscles of control mice, whereas a large number of EGFP^+^ cells infiltrated in the ischaemic muscle at D3 and D7. Scale bar, 50 µm. (B‐C) Representative image of FACS analysis of the EGFP^+^ cell percentage in peripheral blood and bone marrow from ischaemic *Hdc*‐EGFP mice and control non‐ischaemic *Hdc*‐EGFP mice at D3 and D7. The percentage of EGFP^+^ cells increased in blood and bone marrow, and most of the EGFP^+^ cells were CD11b^+^ myeloid cells. D, Percentage of EGFP^+^ cells in blood and bone marrow. n = 5 per group. E, Immunofluorescence staining of EGFP^+^ cells with CD11b^+^ in injured gastrocnemius muscle from *Hdc*‐EGFP mice at D3 and D7. Most of the EGFP^+^ cells infiltrated in injured muscle (above 90%) were CD11b^+^ myeloid cells. Scale bar, 50 µm. F, Number of EGFP^+^CD11b^+^ cells in injured muscle of *Hdc*‐EGFP mice at D3 and D7 per field of view. n = 5 mice per group. G, Immunofluorescence costaining of the satellite marker pax‐7 (red) and EGFP^+^ cells (green); none were merged. Scale bar, 50 µm. For all experiments, error bars represent the mean ± SD. *P < .05, **P < .01, ***P < .001, ****P < .0001

There is no obvious evidence from previous studies on HDC expression in injured limb muscle. We therefore used *Hdc*‐EGFP transgenic mice (*Hdc*‐EGFP) to establish the ischaemic limb injury model, in which the expression of GFP could help to identify HDC‐expressing cells. The results of immunofluorescence staining showed that few EGFP^+^ cells were distributed in the skeletal interstitium and connective tissue of *Hdc*‐EGFP mice in the control group (Figure 2A). However, a large amount of EGFP^+^ cells was observed in the connective tissue of the ischaemic muscles at day 3 and day 7 after injury (Figure 2A). Flow cytometric analysis (FACS) data also confirmed the significant increases in EGFP^+^ cells in the peripheral blood (Figure 2B) and bone marrow (Figure 2C) of *Hdc*‐EGFP mice at day 3 and day 7 relative to control mice. FACS data further clarified that most EGFP^+^ cells highly expressed the myeloid cell markers CD11b^+^ and Gr‐1^+^ (Figure 2B‐D). In addition, the immunofluorescence staining results with anti‐CD11b confirmed that approximately 90% of EGFP^+^ cells in the ischaemic injured muscle were CD11b^+^ myeloid cells (Figure 2E,F).

We examined whether myoblast and muscle cells express HDC. The immunofluorescence staining results with the SC marker PAX‐7 revealed that no EGFP expression was present in these PAX‐7^+^ SCs of *Hdc*‐EGFP mice (Figure 2G). To investigate whether HDC‐expressing cells derived from the bone marrow and spleen reservoirs are capable of converting into skeletal muscle, *Hdc*‐CreERTM:Rosa26mTmGFP(*Hdc*‐CreERTM‐mTmGFP) mice were used to establish a limb ischaemia model to trace HDC‐expressing stem cells or progenitors and their descendants (the diagram is shown in Figure S2A). After induction by tamoxifen injection, many mTmGFP^+^ cells could be observed in the interstitial connective tissue of the injured limb at day 3, day 7 and day 14, but we failed to detect the expression of mTmGFP in skeletal muscle and myoblasts (Figure S2B). FACS data provided evidence that the percentage of mTmGFP^+^ cells significantly increased in the blood, spleen and bone marrow at day 3 post‐injury and decreased at day 7 (Figure S2C). In addition, FACS data demonstrated that mTmGFP is highly expressed in CD11b^+^ myeloid cells (approximately 90%) and CD11b^+^Ly6C^+^ monocytes (approximately 50%) in the peripheral blood of *Hdc*‐CreERTM‐mTmGFP mice (Figure S2D‐E). Taken together, these data demonstrate that HDC is expressed predominantly in CD11b^+^ myeloid cells but not in skeletal muscle or myoblast cells.

### 3.3 Histamine deficiency induces aberrant inflammation and muscle fibre damage

**Figure 3:**
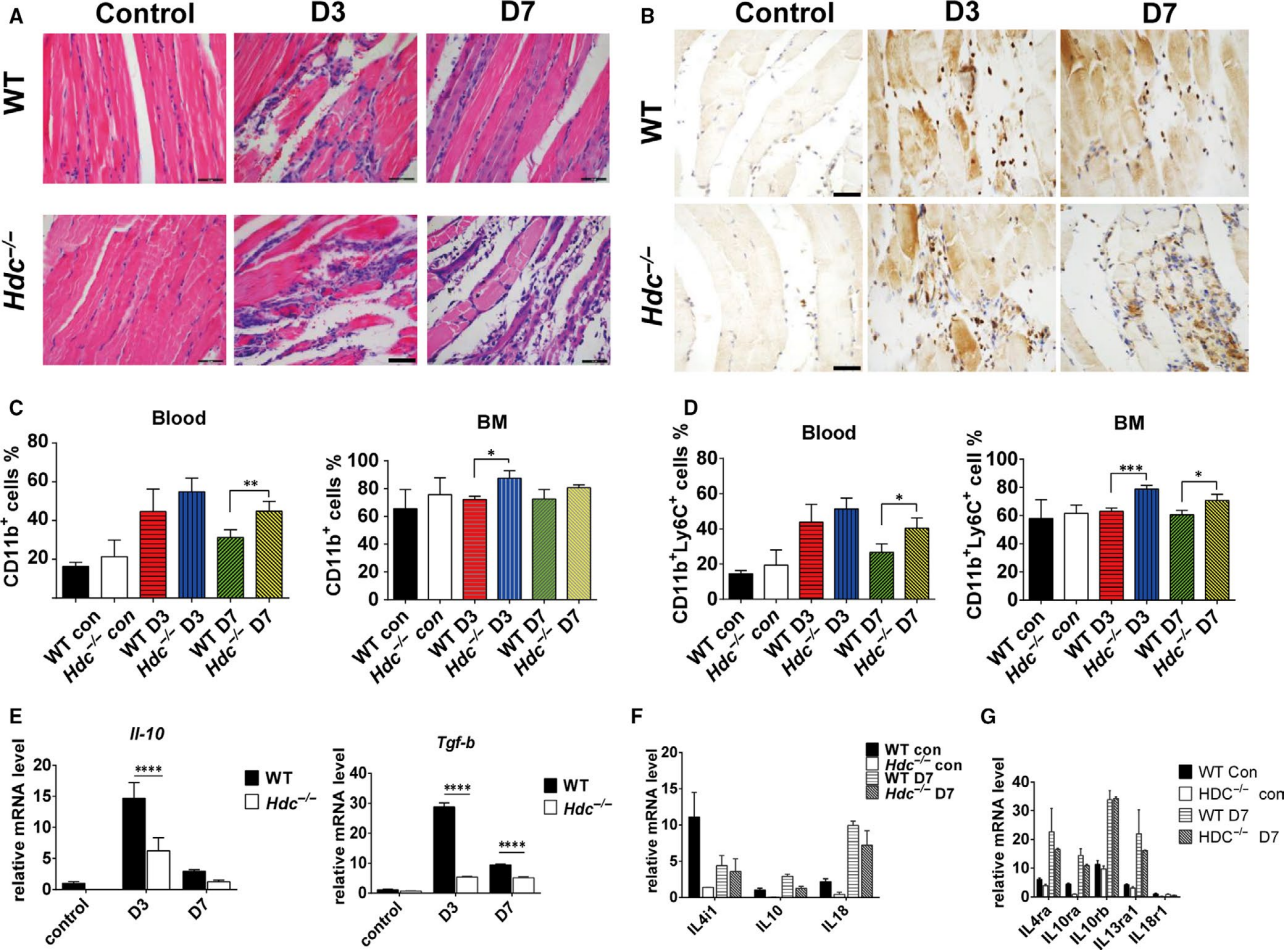
Histamine deficiency induces an aberrant immune response and degenerates muscle fibres after injury. A, Haematoxylin and eosin (H&E) staining of injured gastrocnemius muscle from *Hdc^−/−^*and WT mice at D3 and D7 post‐injury. Significant myofibre necrosis, smaller muscle fibres and inflammatory lesions were observed in *Hdc^−/−^*mice relative to WT mice. Scale bar, 50 µm. B, Anti‐CD11b immunohistochemistry staining of injured gastrocnemius muscle from *Hdc^−/−^* and WT mice at D3 and D7 post‐injury. In both groups, plenty of CD11b^+^ cells were seen at D3, but lots of CD11b^+^ cells still could be found in injured muscles of *Hdc^−/−^* mice at D7. Scale bar, 50 µm. (C‐D) FACS analysis of the relative proportion of CD11b^+^ myeloid cells and CD11b^+^Ly6C^+^ monocytes in the peripheral blood and bone marrow at D3 and D7 after limb ischaemia. n = 8 mice per genotype. E, qRT‐PCR analysis of the mRNA expression of the anti‐inflammatory cytokines *Il‐10* and *Tgf‐β* in ischaemic muscle from *Hdc^−/−^* and WT mice at D3 and D7 post‐injury. n = 3 biological replicates. (F‐G) mRNA expression of cytokines and cytokine receptors was tested in the ischaemic muscles from *Hdc^−/−^*and WT mice at D7 after ischaemia by RNA‐Seq analysis. n = 3 biological replicates. For all experiments, error bars represent the mean ± SD. *P < .05, **P < .01, ***P < .001, ****P < .0001

Morphological analysis of muscles at day 7 post‐injury revealed that degenerating and necrotic muscle fibres were largely cleared in WT mice and replaced by multinucleated myoblasts, indicating the early‐phase of muscle regeneration (Figure 3A top). Consistent with the results mentioned above, we observed that histamine deficiency in *Hdc^−/−^* mice seems to cause chronic inflammation and delay wound healing, as demonstrated by quite a large number of immune cells, necrotic fibres, loss of muscle fibre integrity and smaller muscle fibres in ischaemic muscle compared to those in WT mice at day 7 post‐injury (Figure 3A). Given that the immune response plays an important role in muscle ischaemic injury and wound healing, the expression of immune cells and inflammatory cytokines was examined in *Hdc^−/−^* and WT mice with ischaemic injury. The results of H&E staining showed that the tissue injury and inflammatory reaction were more severe in *Hdc^−/−^*mice than in WT mice (Figure 3A). At day 3 and day 7, HE staining results indicated that many more immune cells had infiltrated throughout the injured muscle, and significant myofibre necrosis was observed in the injured limbs of *Hdc^−/−^*mice relative to WT mice (Figure 3A). The immunohistochemistry staining results with anti‐CD11b demonstrated that the majority of immune cells highly expressed CD11b at day 3 and day 7 post‐injury (Figure 3B). FACS data showed that the percentage of CD11b^+^ myeloid cells and CD11b^+^Ly6C^+^ monocytes markedly increased in the peripheral blood and bone marrow of *Hdc^−/−^* mice and WT mice at day 3 and day 7 after limb ischaemia (Figure 3C‐D). Interestingly, we observed higher expression levels of CD11b^+^ myeloid subsets in *Hdc^−/−^* mice than in WT mice during the whole reaction process, particularly at day 7 post‐injury (Figure 3C‐D). In addition, the results of quantitative RT‐PCR showed that the mRNA levels of the anti‐inflammatory cytokines *Il‐10* and *Tgf‐β* were markedly decreased in the muscles of *Hdc^−/−^* mice relative to WT mice (Figure 3E). The results of RNA‐Seq analysis of ischaemic muscle suggested that mRNA expression levels of some anti‐inflammatory cytokines, cytokine receptors, chemokines and chemokine receptors were significantly decreased in the injured muscles of *Hdc^−/−^*mice relative to WT mice (Figure 3F‐G, Figure S3A‐B). Taken together, these data indicate that histamine deficiency induces a disordered pattern of systemic inflammation and immune responses, leading to the augmentation of skeletal muscle injury and the delay of regeneration.

### 3.4 Histamine deficiency diminishes the proliferation of myoblast cells

**Figure 4:**
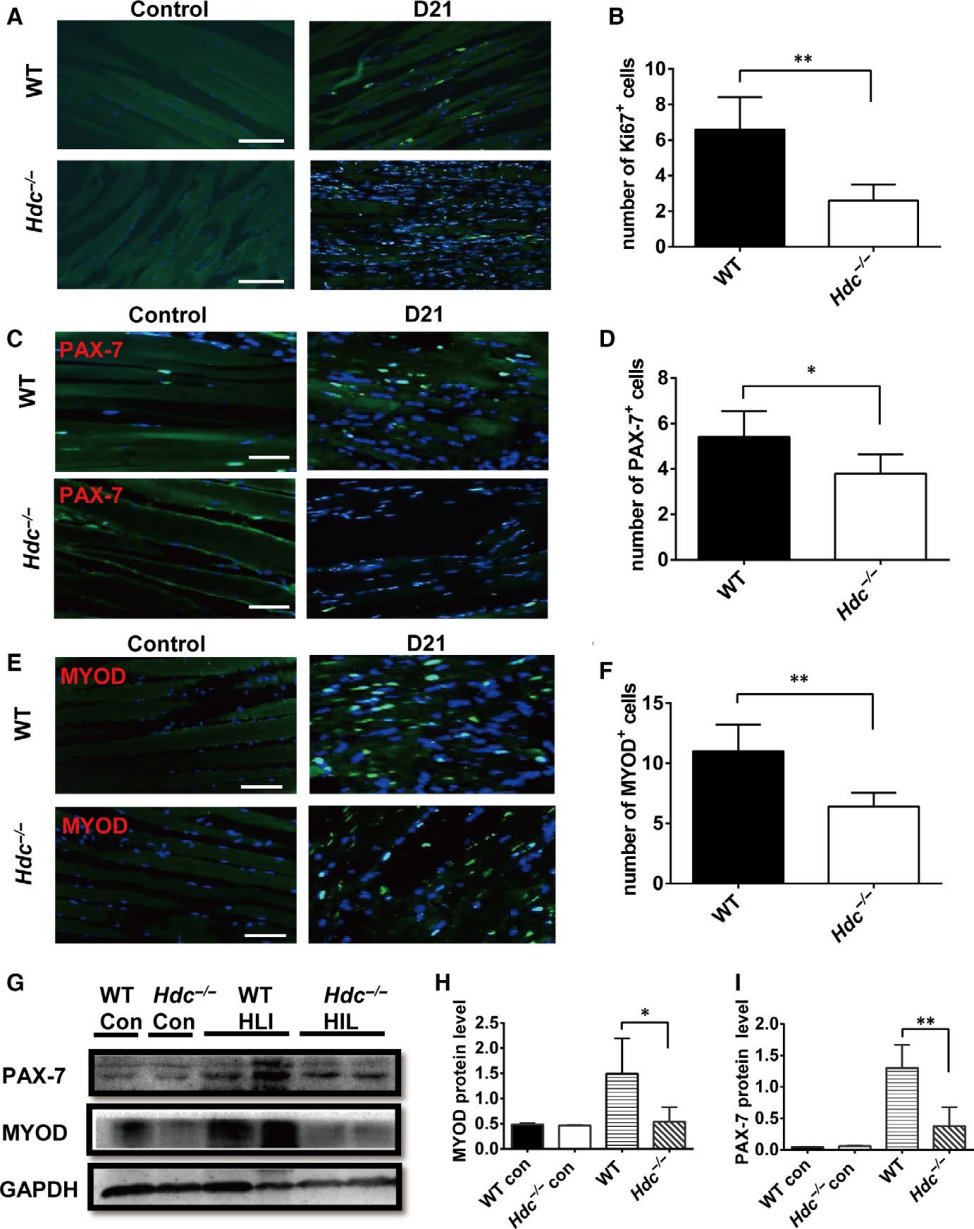
Histamine deficiency represses myoblast proliferation. A, Ki67 immunofluorescence staining in ischaemic gastrocnemius muscle from *Hdc^−/−^*and WT mice at D21 after limb ischaemia. Scale bar, 50 µm. B, Number of Ki67^+^cells per field of view. The number of Ki67^+^ cells was significantly decreased in the muscles of *Hdc^−/−^*mice. **P < .01. n = 5 mice per genotype. C, Immunofluorescence staining with the myoblast marker Pax‐7 in injured gastrocnemius muscle from *Hdc^−/−^*and WT mice at D21 post‐injury. D, Number of PAX‐7^+^ cells per field of view. The number of PAX‐7^+^ cells was significantly decreased in the muscles of *Hdc^−/−^*mice. **P < .01. n = 5 mice per genotype. E, Immunofluorescence staining of injured gastrocnemius muscle from *Hdc^−/−^*and WT mice at D21 post‐injury with the myoblast marker MYOD. F, Number of MYOD^+^ cells per field of view. The number of MYOD^+^ cells was significantly decreased in the muscles of *Hdc^−/−^*mice. *P < .05. n = 5 mice per genotype. G, Western blot analysis of PAX‐7 and MYOD in injured muscle from *Hdc^−/−^*and WT mice at D21 post‐injury. The results from two independent preparations are shown. (H‐I) Protein levels of Pax‐7 and MYOD in injured muscle at D21 were quantified by Western blot assay. Both Pax‐7 and MYOD protein levels were decreased in the ischaemic muscle of *Hdc^−/−^*mice. *P < .05, **P < .01. n = 5 mice per genotype. Data were quantified by ImageJ. Data are represented as the mean ± SD

Given that satellite stem cells (SCs) are a well‐characterized population of self‐renewing regenerative cells, and regeneration of new myofibres occurs in the skeletal muscle tissue, we investigated the effect of histamine on the proliferation of SCs. The IHC results with the cell proliferation marker anti‐Ki67 (Figure 4A) at day 21 showed that the numbers of proliferating cells in the ischaemic muscle were significantly less in the *Hdc^−/−^* group relative to the WT group (Figure 4B). Quiescent SCs are marked by transcription factors of the paired homeodomain family PAX‐7, and SC cells, which re‐enter the cell cycle and activate the myogenic process, express MYF5 and MYOD. Immunofluorescence staining data demonstrated that the numbers of PAX‐7^+^ SC cells (Figure 4C) in the *Hdc^−/−^* group and the proliferating stage SC cells marked by MYOD (Figure 4E) in the *Hdc^−/−^* group were significantly decreased relative to the WT group (Figure 4D,F). In addition, the Western blot results confirmed the down‐regulation of the PAX‐7 and MYOD proteins in the *Hdc^−/−^* group relative to the WT group (Figure 4G‐I).

**Figure 5:**
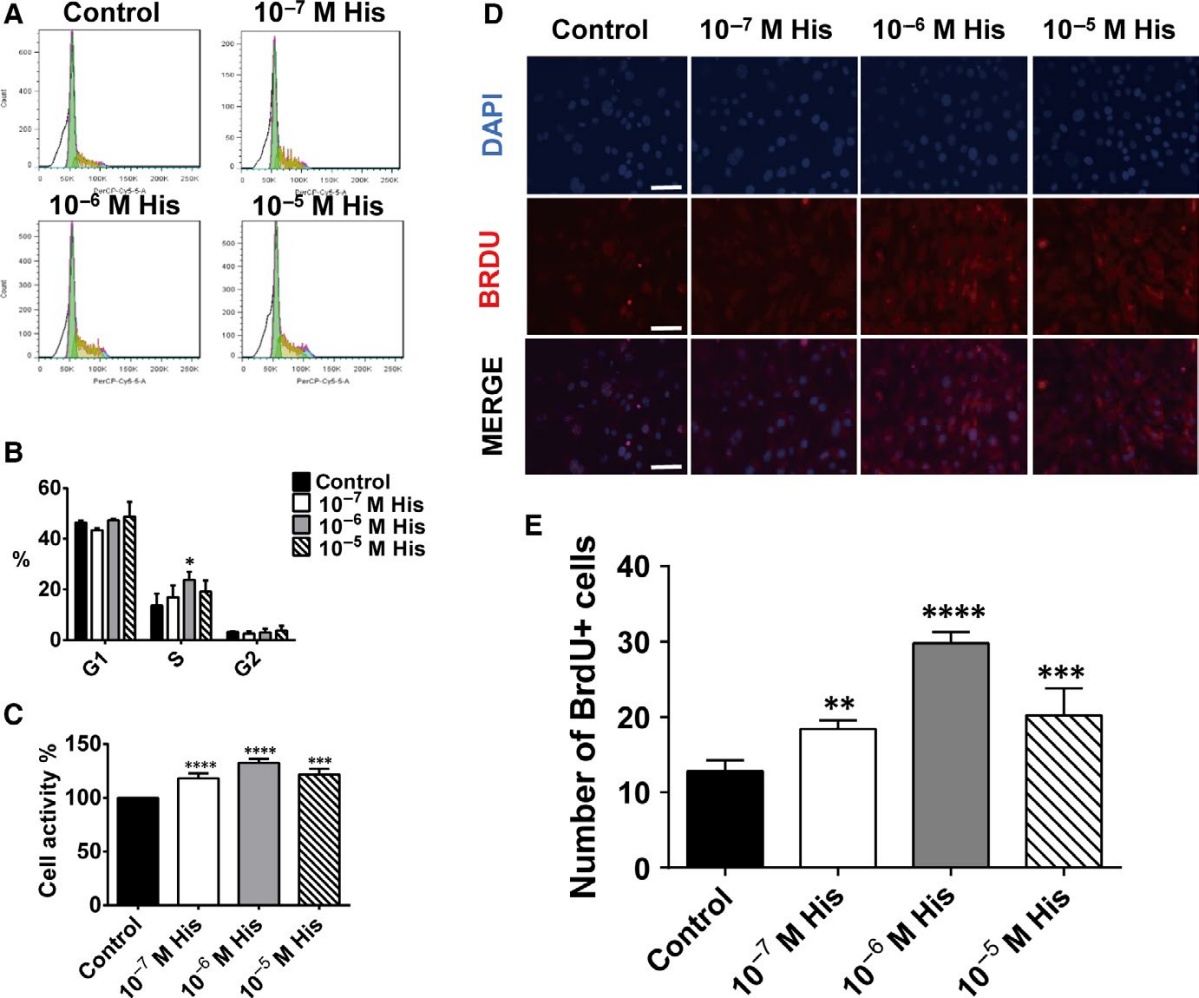
Histamine promotes the proliferation of C2C12 cells. A, Representative image of FACS cell cycle examination of C2C12 cells given different concentrations of histamine. B, Percentage of S‐stage cells. S‐stage cells were increased after histamine was given, and the increase was most significant at a histamine concentration of 10^−6^ mol. n = 3. C, CCk8 cell proliferation test after the administration of different concentrations of histamine. When given histamine, the cells became more proliferative. n = 5. D, Cell proliferation test with BrdU immunofluorescence staining. Scale bar, 25 µm. E, Number of BrdU+ cells per field. Given 10^−6^ mol histamine, cells became more proliferative. n = 3. For all experiments, error bars represent the mean ± SD. *P < .05, **P < .01, ***P < .001, ****P < .0001

Furthermore, the C2C12 myoblast cell line was used to validate the effects of histamine on myoblast proliferation. Different concentrations of histamine were administered in C2C12 myoblast cell culture, and the results of cell cycle analysis showed that a moderate concentration of histamine (10^−6^ mol/L) significantly increased the percentage of proliferating C2C12 myoblast cells (cell cycle S phase) (Figure 5A,B). In addition, the results of a CCK8 cell proliferation assay (Figure 5C) and anti‐BrdU staining (Figure 5D,E) also confirmed the effect of a moderate concentration of histamine on the proliferation of C2C12 myoblast cells.

### 3.5 Histamine increases IGF‐1 levels

**Figure 6:**
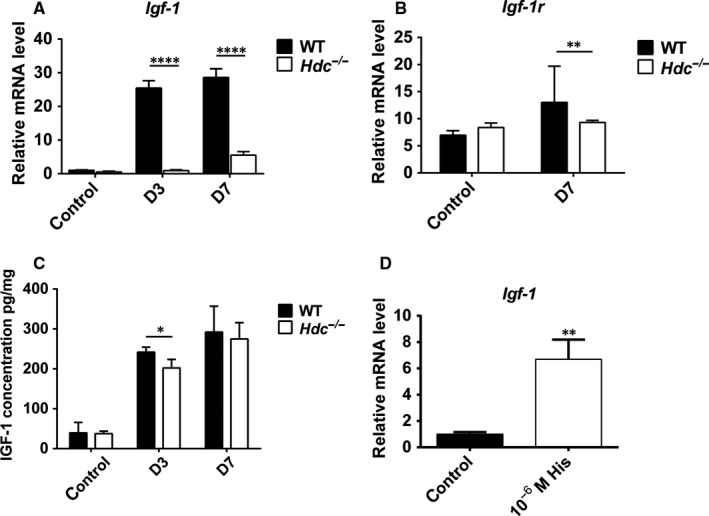
Histamine increase IGF‐1 levels. A, *Igf‐1* mRNA levels in ischaemic muscle of *Hdc^−/−^*and WT mice at D3 and D7 post‐injury were quantitated by qRT‐PCR. n = 3 biological replicates. B, *Igf‐1* receptor (*Igf‐1r*) mRNA levels in ischaemic muscles of *Hdc^−/−^*and WT mice at D7 post‐injury were quantitated by qRT‐PCR. n = 3 biological replicates. C, The IGF‐1 concentration in ischaemic muscle of *Hdc^−/−^*and WT mice at D3 and D7 post‐injury was tested by ELISA assay. n = 5 biological replicates. D, *Igf‐1* mRNA levels in C2C12 cells were quantitated by qRT‐PCR after treatment with histamine for 2 h. n = 3. For all experiments, error bars represent the mean ± SD. *P < .05, **P < .01, ***P < .001, ****P < .0001

It is well known that insulin‐like growth factor (IGF‐1) is a critical growth factor for many tissues, especially skeletal muscle growth. The qRT‐PCR results showed that *Igf‐1* mRNA levels were decreased in the skeletal muscle of *Hdc^−/−^* mice relative to WT mice at day 3 and day 7 post‐injury (Figure 6A). Down‐regulation of IGF‐1 receptor (*Igf‐1r*) mRNA levels was also observed in the injured muscles of *Hdc^−/−^*mice compared to WT mice at day 7 post‐injury (Figure 6B). Normally, *Igf‐1r* expression increases significantly after skeletal muscle injury, which was observed in the injured muscle of WT mice. However, *Igf‐1r* mRNA levels did not increase in the injured muscle of *Hdc^−/−^* mice relative to control *Hdc^−/−^* mice (Figure 6B). Furthermore, we examined the concentration of IGF‐1 in the injured muscles of *Hdc^−/−^* and WT mice post‐injury by ELISA. The results confirmed the expression levels of IGF‐1 decreased in the injured muscles of *Hdc^−/−^* mice compared with those of WT mice at day 3 and day 7 post‐injury (Figure 6C). In addition, in vitro cell culture results confirmed that incubation with exogenous histamine (10^‐6^ mol/L) significantly increased *Igf‐1* mRNA levels in C2C12 cells (Figure 6D). These results indicated that histamine increases the expression level of IGF‐1 and maintains the activation of the IGF‐1 receptor in response to increased IGF‐1 in ischaemic muscle.

### 3.6 Histamine increases C2C12 myoblast cell proliferation through H3R/PI3K/AKT signalling

**Figure 7:**
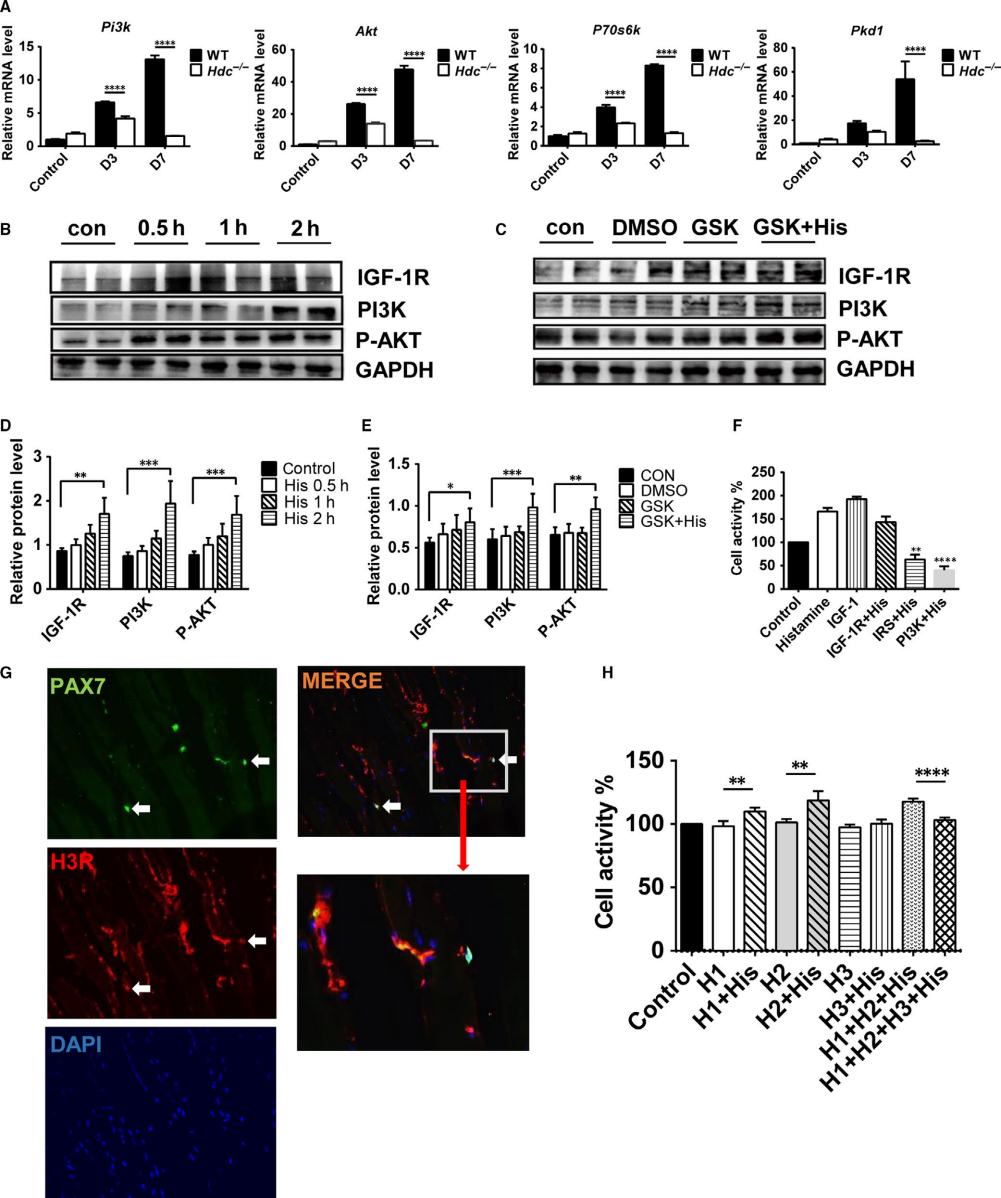
Histamine promotes C2C12 cell proliferation by activating H3R and the IGF‐1/IGF‐R/PI3K/AKT signalling pathway independently. A, The mRNA expression levels of the IGF‐1/PI3K/AKT pathway downstream molecules *Pi3k*, *Akt*, *Pkd1* and *P70s6k* in ischaemic muscles of *Hdc^−/−^*and WT mice at D3 and D7 post‐ischaemia were tested by qRT‐PCR. The results showed decreased gene expression of the downstream signalling molecules in the ischaemic muscle of *Hdc^−/−^*mice. n = 3 biological replicates. B, Western blot analysis of IGF‐1R, PI3K (p85), P‐AKT and GAPDH protein expression in C2C12 cells after treatment with histamine. The results from two independent preparations are shown. C, Western blot analysis of IGF‐1R, PI3K (p85), P‐AKT and GAPDH protein expression in C2C12 cells blocking IGF‐R by an antagonist (GSK1904529A, GSK). The results from two independent preparations are shown. D, Protein levels of IGF‐1R, PI3K (p85), P‐AKT and GAPDH in C2C12 cells after histamine treatment were quantified by Western blot assay. Histamine increased IGF‐1R, PI3K (p85) and P‐AKT protein expression levels in C2C12 cells. n = 6. E, Protein levels of IGF‐1R, PI3K (p85), P‐AKT and GAPDH in C2C12 cells after blocking IGF‐R by an IGF‐1R antagonist were analysed by Western blot assay. After blocking the IGF‐1/IGF‐R/PI3K/AKT signalling pathway, histamine treatment still increased IGF‐1R, PI3K (p85) and P‐AKT protein expression levels in C2C12 cells. n = 6. F, CCk8 cell proliferation test of C2C12 cells treated with histamine, IGF‐1, IGF‐1R antagonist (GSK1904529A) & histamine, IRS antagonist (NT157) & histamine, and PI3K antagonist (LY294002) & histamine. The PI3K antagonist significantly blocked the histamine effect on C2C12 cell proliferation. n = 5. G, Immunofluorescence staining of histamine H3 receptor and PAX‐7 in gastrocnemius muscle. Scale bar, 100 µm. H, CCk8 cell proliferation test following treatment of C2C12 cells with histamine receptor (HR) antagonist and histamine (H1R antagonist, H2R antagonist, H3R antagonist, H1R antagonist & histamine, H2R antagonist & histamine, H3R antagonist & histamine, H1R+H2R antagonists & histamine, H1R+H2R+H3R antagonists & histamine). The H3R antagonist significantly blocked the histamine effect on C2C12 cell proliferation. n = 5. For all experiments, error bars represent the mean ± SD. Western blot quantification was performed by ImageJ. *P < .05, **P < .01, ***P < .001, ****P < .0001

The activation of the PI3K/AKT pathway is an important intracellular signal for IGF‐1/IGF‐1R‐mediated regulation of the development and regeneration of skeletal muscle. Given that we found that the expression of *Igf‐1* and *Igf‐1* receptor mRNA decreased in the injured muscle of *Hdc^−/−^* mice relative to WT mice, we aimed to clarify the relationship between the histamine/HR pathway and the IGF‐1R/PI3K/AKT pathway during muscle regeneration. First, mRNA expression levels of IGF‐I/PI3K/AKT pathway components were examined, and the results showed that mRNA levels of *Pi3k*, *Akt*, *Pkd1* and *P70s6k* were decreased in the ischaemic muscles of *Hdc^−/−^* mice at day 3 and day 7 post‐ischaemia compared with those of WT mice (Figure 7A). Subsequently, C2C12 cells were treated with histamine (10^−6^ mol/L) in vitro, and the protein levels of IGF‐1R, PI3K (P85) and P‐AKT were examined by Western blot assay. The results confirmed that protein levels of IGF‐1R, PI3K (P85) and P‐AKT were elevated significantly in C2C12 cells in response to histamine incubation (Figure 7B,D). However, when we blocked the IGF‐1/IGF‐1R pathway with an IGF‐1R antagonist (GSK1904529A), histamine could still increase the expression levels of PI3K (P85) and the phosphorylation of AKT (Figure 7C,E). This suggested that histamine activates the intracellular PI3K/AKT pathway, which is not dependent on the activation of the IGF‐1 receptor. We made further efforts to identify the possible downstream signals regulated by histamine in cultured C2C12 cells using an IGF‐1R antagonist (GSK1904529A), IRS antagonist (NT157) and PI3K antagonist (LY294002). The results of a CCK8 cell proliferation assay demonstrated that the IGF‐1R antagonist did not block the histamine proliferative effect, but the IRS antagonist and PI3K antagonist blocked the effect of histamine on C2C12 cell proliferation (Figure 7F). The immunofluorescence staining results indicated that muscle fibres and myogenic cells expressed the histamine H3 receptor (Figure 7G). Histamine H3 receptor mRNA levels were tested in skeletal muscle, CD11b^+^ myeloid cells, isolated satellite cells (SC cells), the neuroglioma cell line U87 and mouse brain tissue. The results demonstrated the mRNA expression of histamine H3 receptor in muscle fibre and myogenic cells (Figure S4A). Thus, we tested different histamine receptor antagonists against histamine receptors H1R, H2R and H3R. The results of a CCK8 cell proliferation test found that histamine activates the PI3K/AKT pathway via the H3 receptor (Figure 7H).

**Figure 8:**
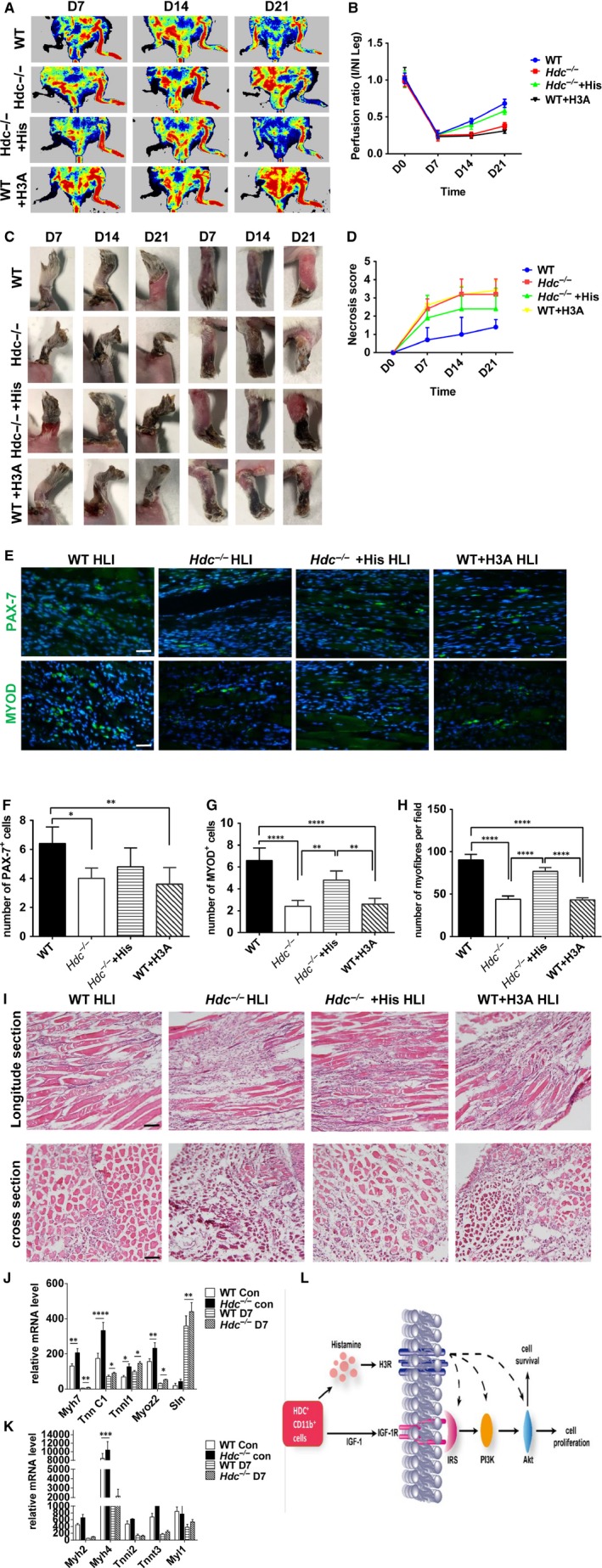
Histamine H3 receptor antagonist delays blood perfusion and functional recovery in ischaemic limbs of WT mice. A, Laser Doppler scanning images at 7, 14 and 21 days after femoral ligation. B, Ischaemic limb perfusion quantitation was analysed by the ratios of blood flow in the ischaemic limb to that in the non‐ischaemic limb at each time‐point. n = 5 mice per genotype. C, A picture of the ischaemic limb is shown at each point per group. Each photograph was taken under the same conditions. D, The necrosis score of the ischaemic limb was assessed by the range of necrosis. Scoring criteria are available in the Methods section. n = 5 mice per genotype. E, Immunofluorescence staining of PAX‐7 and MYOD in injured gastrocnemius muscle from WT, *Hdc^−/−^*, WT + H3A and *Hdc^−/−^*+histamine mice at day 21 post‐injury. Scale bar, 50 µm. F, Number of PAX‐7^+^ cells per field of view. n = 5 mice per genotype. G, Number of MYOD^+^ cells per field of view. n = 5 mice per genotype. H, The number of fibres per muscle was counted at D21. I, Haematoxylin and eosin (H&E) staining of injured gastrocnemius muscle from WT, *Hdc^−/−^, *WT + H3A and *Hdc^−/−^*+histamine mice at day 21 post‐injury. Scale bar, 100 µm. (J‐K) Fast and slow fibre‐specific genes were tested in injured muscles of *Hdc^−/−^* and WT mice at D7 after ischaemia by RNA‐Seq analysis. n = 3 biological replicates. L, Diagram depicting the mechanism underlying the proliferative role of histamine in myoblasts. For all experiments, error bars represent the mean ± SD. *P < .05, **P < .01, ***P < .001, ****P < .0001

To verify the results showing that histamine promotes C2C12 cell proliferation through the histamine H3 receptor in vitro, an H3R antagonist (H3A) was injected locally into the injured muscle of WT mice after HLI. The perfusion analysis results revealed the significantly delayed recovery of blood flow in ischaemic limbs of WT + H3A mice compared to WT mice at 7, 14 and 21 days post‐injury, which phenocopies the *Hdc^−/−^* mice (Figure 8A,B). Consistent with a low perfusion ratio, a high degree of limb necrosis was observed in WT + H3A mice relative to WT mice (Figure 8C,D). To demonstrate the protective effect of histamine on the injured limb, exogenous histamine was administered in *Hdc^−/−^* mice before and after the surgical operation. As we expected, the administration of exogenous histamine in *Hdc^−/−^* mice significantly improved the ratio of blood perfusion (Figure 8A,B) and repressed the necrosis of the injured limb, almost reaching the level of the WT group (Figure 8C,D). Immunofluorescence staining data demonstrated that the numbers of PAX‐7^+^SC cells and MYOD^+^ proliferating stage SC cells in the WT + H3A group were significantly decreased relative to the WT group (Figure 4E). Exogenous histamine treatment in *Hdc^−/−^* mice increased the number of PAX‐7^+^SC cells (Figure 8F) and MYOD^+^ proliferating stage SC cells (Figure 8G). The number of fibres per muscle was counted, and fewer fibres were seen in the WT + H3A group and *Hdc^−/−^*group at D21 (Figure 8H). The H&E staining results showed that the tissue injury were more severe in the WT + H3A group relative to the WT group, and exogenous histamine treatment in *Hdc^−/−^* mice alleviated tissue injury (Figure 8I). Additionally, the myofibre type gene expression was significantly altered in *Hdc^−/−^* muscle. RNA‐Seq data showed that the expression levels of slow fibre‐specific genes were up‐regulated in injured muscle of *Hdc^−/−^*mice at D7 after ischaemia relative to WT mice (Figure 8J,K).

## 4 DISCUSSION

In this study, we show that histamine deficiency (*Hdc^−/−^*) interrupts the regenerative microenvironment leading to impaired muscle regeneration. *Hdc^−/−^* mice exhibit delayed blood perfusion of the limb with increased necrosis after limb ischaemic injury. Histamine deficiency promotes the recruitment of CD11b^+^ myeloid cells to the sites of injury and represses macrophage differentiation, leading to the hampered proliferation of myoblast cells. Additionally, histamine increases the expression level of IGF‐1 in ischaemic muscles and acts as a co‐factor that activates the PI3K/AKT signalling pathway in C2C12 myoblast cells via the histamine H3 receptor, which is critical for muscle growth and tissue regeneration. Thus, these data reveal a critical role for HDC‐expressing CD11b^+^ myeloid cells and histamine in the proliferation of myoblast cells and tissue regeneration.

It is generally assumed that mast cells are the main source of histamine release in response to injury and wound healing.^27^, ^28^, ^29^, ^30^ However, using *Hdc*‐EGFP transgenic mice, previous studies revealed that CD11b^+^Gr‐1^+^ myeloid cells were the major HDC‐expressing cells in inflammation‐associated tumorigenesis^19^ and myocardial infarction.^20^, ^31^ This suggests that mast cells in tissues may play a role in the temporary storage of histamine derived from these HDC‐expressing cells, such as basophils and CD11b^+^Gr‐1^+^ myeloid cells. In the current study, we not only used Hdc‐EGFP mice to identify the HDC‐expressing cells and the main cellular source of histamine in limb ischaemia but also further clarified whether skeletal muscle cells and myoblasts expressed HDC using Hdc‐CreERTM: Rosa26mTmGFP mice. Consistent with our previous studies, we found that most of the HDC^+^ cells that infiltrated into the ischaemic muscle were CD11b^+^ myeloid subsets, and these cells were mobilized from the bone marrow and spleen. Using *Hdc*‐CreERTM: Rosa26mTmGFP mice to trace HDC‐expressing stem cells or progenitors, the results of lineage tracing demonstrated that no HDC is expressed in PAX‐7^+^ SCs, myoblasts or myofibres. In addition, our data suggested that bone marrow–derived HDC‐expressing CD11b^+^ immune cells cannot be induced to transform into skeletal muscle or myoblasts. A recent study reported that prolonged muscular work (PMW) induces HDC expression in the endomysium and around blood vessels and some muscle fibres, using anti‐HDC IHC staining.^32^ PMW requires a large supply of O_2_ and induces hypoxia in the tissue, which might cause slight inflammation in skeletal muscle and further recruit HDC‐expressing CD11b^+^ myeloid cells.

Tissue regeneration of ischaemic muscle injury involves inflammation, angiogenesis and stem cell proliferation. Any of these three phases of tissue healing is crucial to ischaemic muscle repair. In the early‐phase of inflammation, neutrophils and M1‐type macrophages control the maintenance of the niche and support wound healing through the production of pro‐inflammatory cytokines. After the pro‐inflammatory phase, the anti‐inflammatory phase maintains the tissue repair process, promoting neovascularization and stimulating satellite cell proliferation.^33^, ^34^, ^35^ In this study, mRNA expression levels of the anti‐inflammatory cytokines *Il‐10* and *Tgf‐β* and their receptors were reduced in ischaemic muscle of *Hdc^−/−^* mice relative to WT mice. Following injury, pro‐inflammatory and anti‐inflammatory monocytes/macrophages sequentially occur in injured muscle, and any macrophage phenotype alteration might result in impaired muscle regeneration.^8^ In the current study, we found that CD11b^+^F4/80^+^ macrophages and the expression of chemokines such as Ccl2 and Ccr2 were decreased in the peripheral blood of *Hdc^−/−^* mice, which showed impaired macrophage infiltration into the injured muscle relative to WT mice. This is consistent with previous studies showing that mice lacking Ccr2 or Ccl2 present impaired muscle regeneration and macrophage infiltration inducing modified macrophage activation.^36^, ^37^ These results suggest macrophage dysfunction in injured muscle of *Hdc^−/−^* mice, which might explain why *Hdc^−/−^* mice suffer from chronic inflammation with quite a large number of immune cells, necrotic fibres and loss of muscle fibre integrity in ischaemic muscle relative to WT mice at day 7 post‐injury. Our data showed a lower level of TGF‐β mRNA in the early stage (D3 and D7) of injured muscle of *Hdc^−/−^* mice relative to WT mice. This result does not seem to be consistent with enhanced fibrosis in *Hdc^−/−^* mice. Our data also indicated that the level of *Tgf‐β* mRNA decreased at D7 relative to D3 in the WT group, but the same level was maintained at D7 relative to D3 in the *Hdc^−/−^* group. Additionally, Xiaodong Mu et al identified that TGF‐β is able to promote C2C12 myoblasts to express stem cell markers, while TGF‐β1 treatment also promoted proliferation‐arrested C2C12 myoblasts to re‐enter S‐phase.^38^ Our data also showed that the numbers of immune cells infiltrated into the injured muscle, peripheral blood and bone marrow of *Hdc^−/−^* mice were increased relative to WT mice. Conversely, immune cells, especially macrophages, in ischaemic muscle of WT mice dissipated after the removal of necrotic tissues, initiating tissue regeneration. This suggests that the inflammatory response of *Hdc^−/−^*mice is characterized by persistent and chronic inflammation, and other factors contributing to fibrosis may also be involved. Chronic inflammation in *Hdc^−/−^*mice could delay the activation and proliferation of myoblasts, which was confirmed by more necrotic muscle debris and inflammatory cells observed in ischaemic muscle of *Hdc^−/−^*mice at day 7 post‐ischaemia. In addition, the RNA‐Seq analysis results from ischaemic muscle suggested that mRNA expression levels of inflammatory cytokine receptors such as *Il‐4rα*, *Il‐10rα*, *Il‐13rα* and the expression of many chemokines and chemokine receptors were also significantly decreased in the injured muscle of *Hdc^−/−^*mice. Previous studies reported that cytokines and cytokine receptors, the deficiency of which impaired muscle regeneration, also play important roles in stem cell growth and differentiation and can recruit myoblasts for muscle growth.^14^, ^39^, ^40^, ^41^, ^42^ Additionally, a previous study reported that histamine has a direct effect on fibroblast proliferation in the ischaemic heart through the histamine/STAT6 signalling pathway in vivo and in vitro.^31^ Whether the dysfunction of CD11b^+^ myeloid cells is related to their differentiation or phagocytic ability remains to be investigated in the future. Taken together, the current study suggests that histamine deficiency induced an aberrant immune response leading to tissue necrosis and delayed the beginning of the tissue regeneration process in the injured muscle of *Hdc^−/−^*mice.

During the process of skeletal muscle regeneration, myoblast proliferation and differentiation are the two most important events by which the body can regenerate new muscle fibres to repair damaged tissue. We observed a significant decrease in the myoblast population in the injured muscle of *Hdc^−/−^*mice, which may account for the impaired repair of muscle injury. However, the basic PAX‐7 and MYOD proteins and number of myoblasts were at the same levels as those in uninjured muscle of *Hdc^−/−^*and WT mice, indicating that the diminished myoblast population in the injured muscle of *Hdc^−/−^*mice resulted from a lower myoblast proliferation rate. A lower myoblast proliferation rate in *Hdc^−/−^*mice may be caused by chronic inflammation in the injured muscle, which is insufficient to remove necrotic debris and activate myoblast proliferation. We investigated whether histamine has a direct effect on myoblast proliferation in vitro using the C2C12 myoblast cell line. The results confirmed that exogenous histamine could increase the proliferation of C2C12 cells. Previous studies showed that skeletal muscle repair and regeneration are mainly dependent on the satellite cell proliferation response to injury and deletion or damage of satellite cells, resulting in impaired regeneration of injured muscle.^1^, ^3^, ^43^ Thus, diminished satellite cell or myoblast cell proliferation in the ischaemic muscle of *Hdc^−/−^*mice might be the major mechanism underlying delayed skeletal muscle regeneration.

Many factors, such as insulin‐like growth factor‐1 (IGF‐1), hepatocyte growth factor (HGF), vascular endothelial growth factor (VEGF), basic fibroblast growth factor (bFGF) and platelet‐derived growth factor (PDGF), have been shown to influence the proliferation and differentiation of myoblasts in muscle regeneration. The activation, proliferation and differentiation of myoblasts are greatly influenced by their microenvironment, which consists of secreted cytokines and chemokines released by immune cells and interstitial cells. Growth factors are the main components of the regenerative microenvironment regulating the muscle regeneration process by controlling the proliferation and differentiation of satellite cells.^44^, ^45^ Among these essential growth factors, IGF‐1 is particularly critical for myoblast proliferation and muscle growth. In our study, we found lower mRNA levels and concentrations of IGF‐1 in the injured muscle of *Hdc^−/−^*mice. In vitro and in vivo studies have shown that IGF‐1 deficiency or low concentrations of IGF‐1 impede satellite cell proliferation and muscle growth.^46^, ^47^, ^48^ There are two forms of IGF‐1, circulating IGF‐1 and local IGF‐1. Circulating IGF‐1 is mostly derived from the liver, but it is also derived from skeletal muscle and adipose tissue, and it acts in an endocrine manner controlled by growth hormone (GH).^49^ The sustained local overexpression of IGF‐1 was shown to promote myofibre regeneration and increase levels of myogenic regulatory factors, but the increase of IGF‐1 in the serum with exogenous administration of GH or IGF‐1 does not effectively stimulate myofibre regeneration.^50^ Our results demonstrated that the serum level of IGF showed no significant difference between *Hdc^−/−^*mice and WT mice, in both the ischaemia and control groups. However, the concentration of local IGF‐1 in the injured muscle was decreased in *Hdc^−/−^*mice. In addition, exogenous histamine could increase significantly the mRNA level of *Igf‐1* in C2C12 cells and promote C2C12 cell proliferation. Thus, histamine could induce local IGF‐1 release to promote myoblast proliferation and further improve muscle regeneration. Meanwhile, we found delayed blood perfusion and decreased VEGF protein levels in ischaemic muscle of *Hdc^−/−^*mice compared to WT mice. VEGF and angiogenesis were demonstrated to stimulate muscle healing by promoting angiogenesis, which is essential to the formation of a regenerative microenvironment for promoting satellite cell proliferation.^51^, ^52^ In addition to being described as an essential growth factor in muscle regeneration, recent studies have reported that IGF‐1 can also regulate immune responses.^15^, ^16^, ^53^ Therefore, an aberrant immune response in ischaemic muscle of *Hdc^−/−^*mice may be due to a low concentration of IGF‐1 or an abnormal immune response, and low concentrations of IGF‐1 commonly lead to impaired muscle regeneration of *Hdc^−/−^*mice.

Local IGF‐1 induces both the proliferation and differentiation of myoblasts, and its regenerative function comes into effect by activating type I IGF receptor (IGF‐IR) and further activates the downstream signalling pathway. We found decreased *Igf‐1r* mRNA levels in injured muscle of *Hdc^−/−^*mice relative to WT mice. We also observed that gene expression of the IGF‐1/PI3K/AKT pathway downstream molecules *Pi3k*, *Akt*, *Pkd1* and *P70s6k* was decreased in ischaemic muscle of *Hdc^−/−^*mice. Incubating C2C12 cells with exogenous histamine increased the protein levels of IGF‐1R, PI3K and AKT, which indicated that histamine could activate the IGF‐1R/PI3K/AKT signalling pathway during the process of muscle regeneration. A recent study showed that IGF‐IR was required for PI3K activation, and PI3K bound to IGF‐IR plays a positive role in IGF‐1‐dependent cell proliferation,^54^ which can explain the decreased *Igf‐1r* expression with IGF‐1/IGF‐IR/PI3K/AKT pathway inactivation in *Hdc^−/−^*mouse muscle regeneration. Interestingly, we observed that histamine still increased C2C12 cell proliferation despite the IGF‐1R/PI3K/AKT pathway being blocked by an IGF‐1R antagonist (GSK1904529A). This suggested that the histamine pathway may undertake crosstalk with the IGF‐1R/PI3K/AKT signalling pathway. This is consistent with other reports showing that histamine increased the growth of glioma cells by inducing the expression of IGF‐1, and blocking the proliferation‐inducing effect of supplemented IGF‐I did not affect histamine‐stimulated proliferation.^23^ We found that the PI3K antagonist blocked the histamine proliferative effect on C2C12 cells, and the histamine H3 receptor antagonist blocked the histamine effect on C2C12 cell proliferation. These results suggest that histamine promotes C2C12 cell proliferation by activating PI3K/AKT via the H3 receptor (H3R). This result is consistent with a previous study, which proved that the inhibition of H3R leads to suppressed cell invasion by inactivating the PI3K/AKT pathway in gliomas.^55^ Apart from the PI3K/AKT pathway, the RNA‐Seq data from injured muscle suggest that the TNF‐a signalling pathway and NF‐kappa B signalling pathway are significantly down‐regulated in injured muscle of *Hdc^−/−^*mice. These signalling pathways might be another candidate mechanism for exploring the effect of histamine on the proliferation of satellite cells and myoblast cells in future studies. Additionally, myofibre type gene expression was significantly altered in *Hdc^−/−^*muscle, which indicates histamine may have an effect on myofibre type formation. These assumptions will be investigated in a future study.

In conclusion, HDC‐expressing CD11b^+^ myeloid cells are the main cellular source of histamine in ischaemic muscle regeneration. Histamine deficiency diminished the proliferation of tissue stem cells, leading to impaired muscle regeneration. Histamine acts as a myogenic factor to induce essential growth factor and cytokine release, which are vital to the formation of a regenerative microenvironment. Histamine increases IGF‐1 levels and activates the PI3K/AKT pathway mainly through the H3 receptor, which promotes cell proliferation and cell survival. The findings in the current study highlight the critical roles of HDC and/or histamine involved in immune surveillance and in protection against ischaemic muscle injury. Our results also suggest novel therapeutic targets for the application of histamine or its downstream agonists in skeletal muscle regeneration.

## CONFLICT OF INTEREST

The authors declare no conflicts of interest.

## AUTHOR CONTRIBUTIONS

MA, ZWH and ZWW were involved in the study design, completion of experiments, data analysis and manuscript preparation. ZZL helped with the operation of the HLI model. DSL helped with cell culture experiments. ZWW helped with animal care and FACS analysis. CJM and LH helped with histological examinations. ZZW helped with data processing. WCS, HT and GJB helped with the revision of the manuscript. XDY designed the study and contributed to the data analysis and writing of the manuscript. All authors discussed the results and commented on the manuscript.

## Data Availability

The processed and normalized data sets supporting the conclusions of this article are included within the article (File S1). Raw data used during the current study are available from the corresponding author upon reasonable request.
