# Supplementary material for: Histamine deficiency delays ischaemic skeletal muscle regeneration via inducing aberrant inflammatory responses and repressing myoblast proliferation
Source: J Cell Mol Med. 2019 Oct 10;23(12):8392–409. doi: 10.1111/jcmm.14720 (PMC6850925; doi:10.1111/jcmm.14720)
Supplement: Supplementary file 1 [file JCMM-23-8392-s001.pdf]

Figure S1

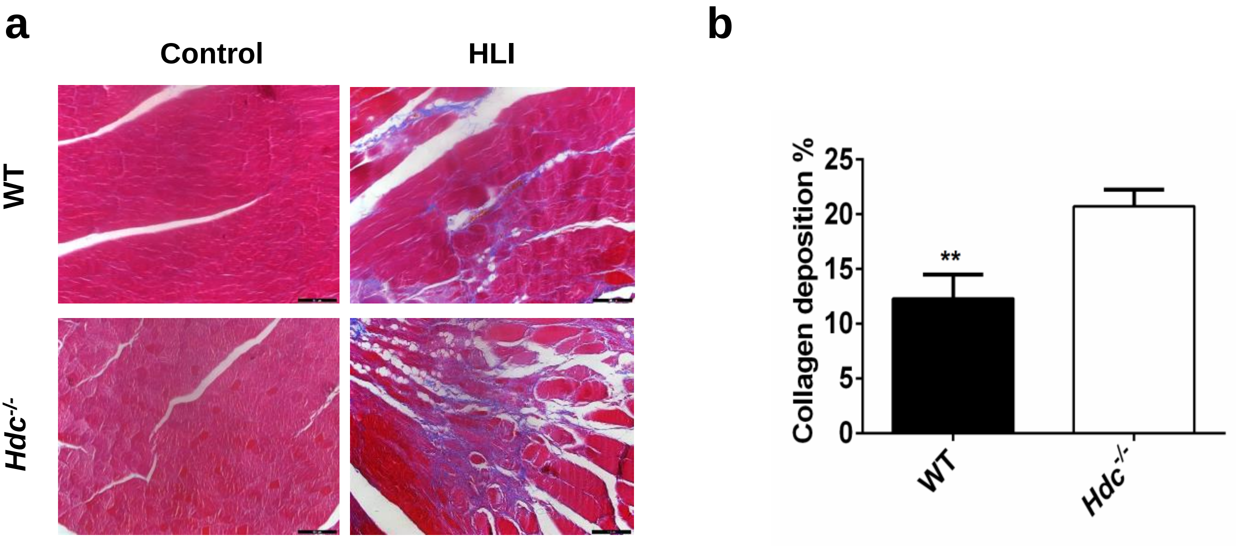

Figure S1. Histamine deficiency delays the blood perfusion and functional recovery in ischemic limbs .

(a) Masson's trichrome staining of injured gastrocnemius muscle from Hdc-/- and WT mice at day21 post-injury. Scale bar, 50 μm. (b) Percentage of collagen deposition at day21 per field of view. n = 8 per genotype. A significant increased interstitial fibrosis was shown in Hdc-/- mice compared with WT mice.

Figure S2

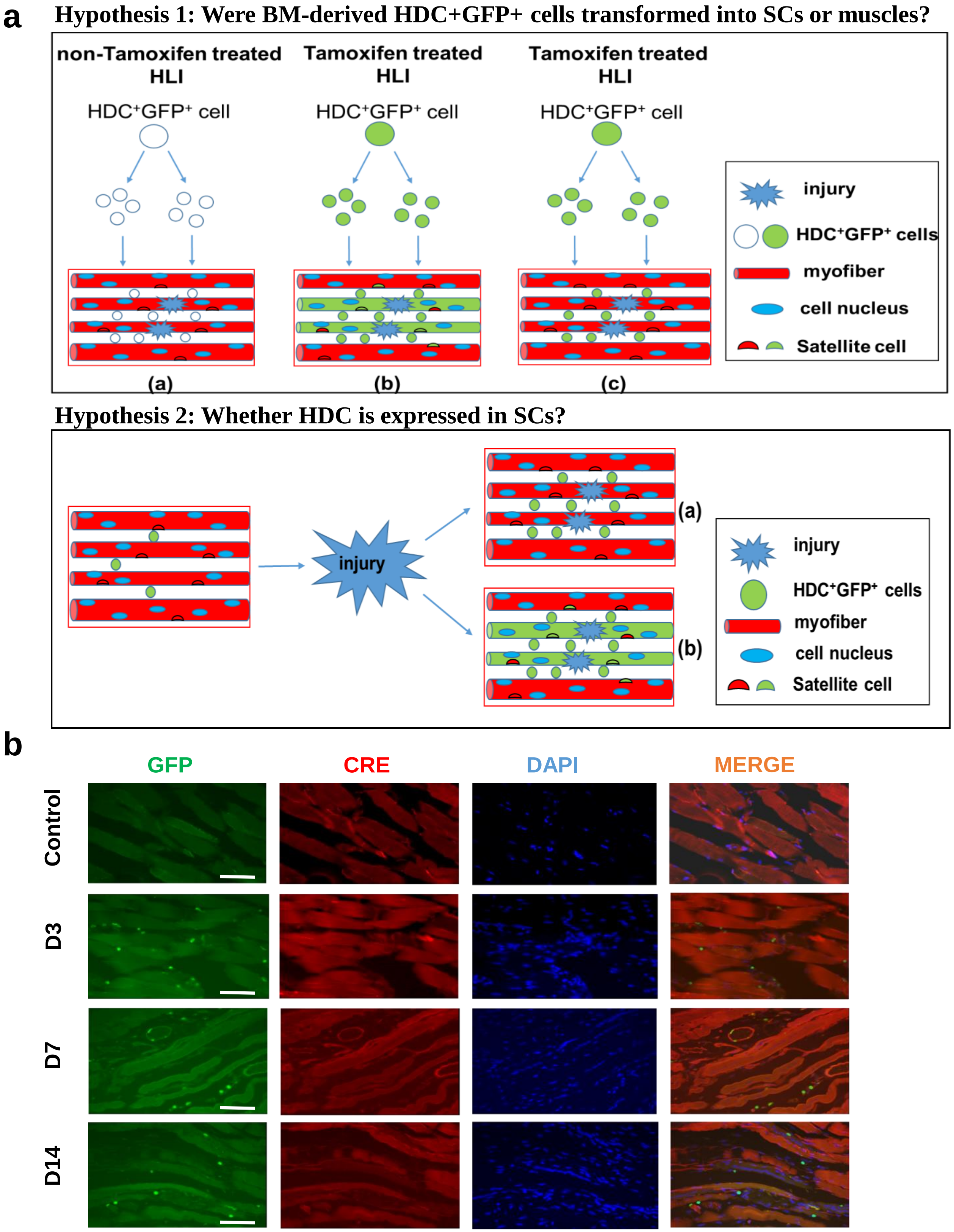
a
Hypothesis 1: Were BM-derived HDC+GFP+ cells transformed into SCs or muscles?
non-Tamoxifen treated HLI
Tamoxifen treated HLI
Tamoxifen treated HLI
HDC⁺GFP⁺ cell
HDC⁺GFP⁺ cell
HDC⁺GFP⁺ cell
(a)
(b)
(c)
injury
HDC⁺GFP⁺ cells
myofiber
cell nucleus
Satellite cell
Hypothesis 2: Whether HDC is expressed in SCs?
injury
(a)
(b)
injury
HDC⁺GFP⁺ cells
myofiber
cell nucleus
Satellite cell
b
GFP
CRE
DAPI
MERGE
Control
D3
D7
D14

c

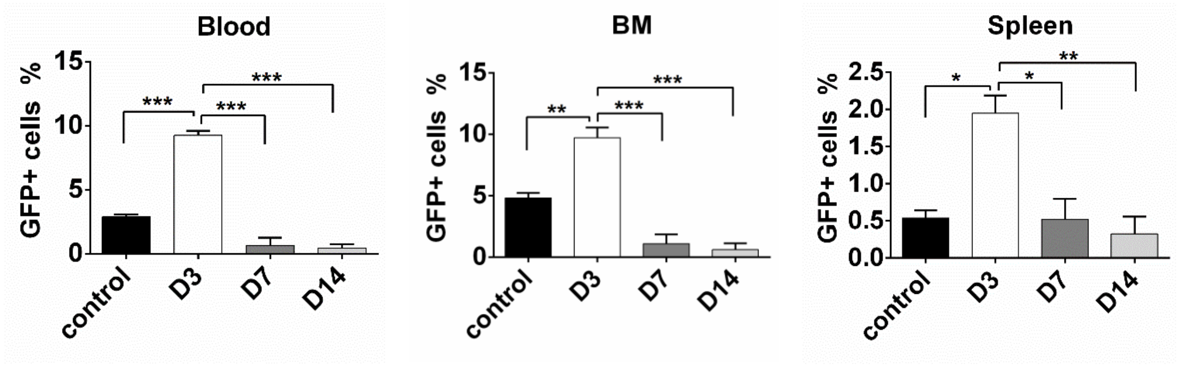
Blood
BM
Spleen
GFP+ cells %
***
**
*
control
D3
D7
D14

d

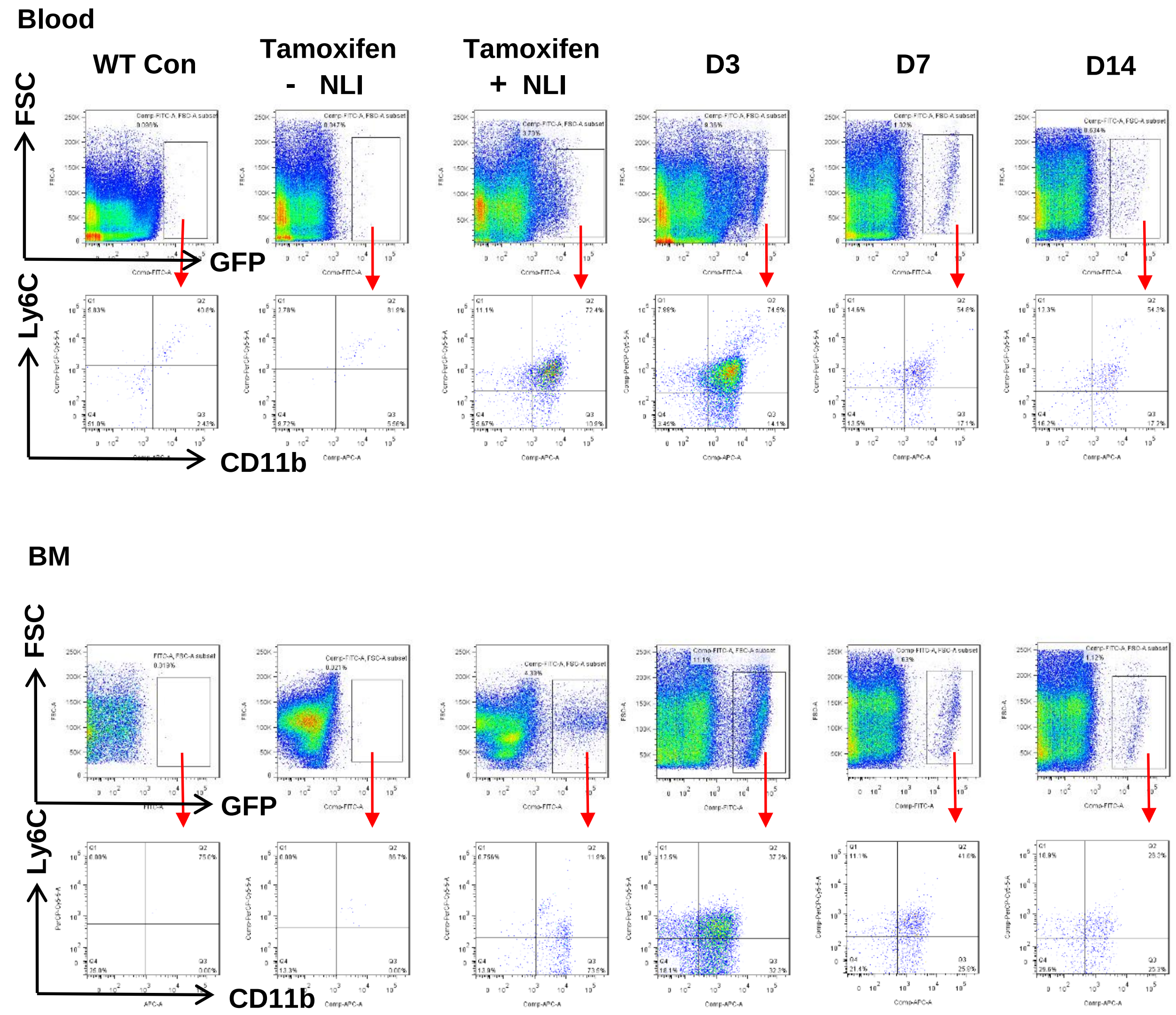
Blood
WT Con
Tamoxifen - NLI
Tamoxifen + NLI
D3
D7
D14
FSC
GFP
Ly6C
CD11b
BM

e

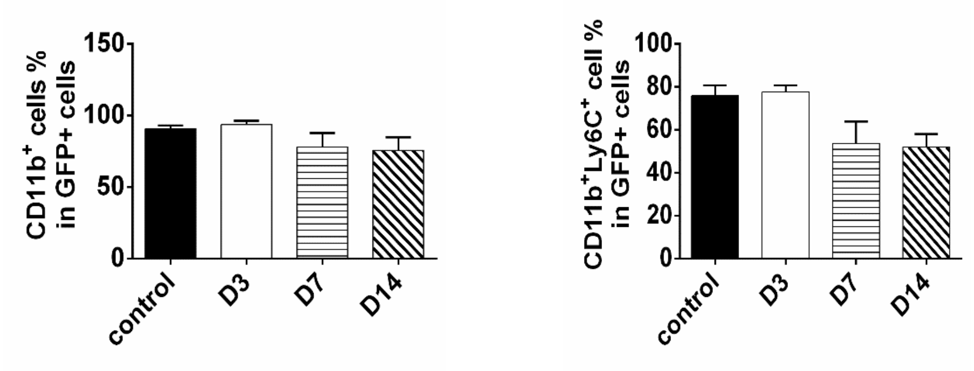

Figure S2. The expression of HDC in the injured limb.

(a) Diagram depicting linage tracing of GFP$^+$ cells from *Hdc*-CreERTM mice in limb ischemia. a: Cells from non-tamoxifen treated *Hdc*-CreERTM mice don't express GFP; b: HDC$^+$GFP$^+$ bone marrow cells differentiate into myoblasts or myofibers and HDC expressed in myoblasts and myofibers; c: HDC$^+$ bone marrow cells just release histamine and don't differentiate into any myoblasts or myofibers, which don't express HDC. From immunofluorescence staining result, we observed none of EGFP expressing HDC$^+$ bone marrow cells can differentiate into myoblasts or myofibers. (b) Immunofluorescence staining ischemic gastrocnemius muscle from tamoxifen treated *Hdc*-CreERTM:Rosa26mTmGFP(*Hdc*-CreERTM) mice at D3, D7, D14 after limb ischemia. GFP$^+$ cells can be seen in the interstitial of the muscle fiber. Neither muscle fibers nor myoblasts derived from GFP$^+$ cells have been seen in the tissue. Scale bar, 50 μm. (c) FACS analysis of relative percentage of GFP$^+$ cells in in blood, bone marrow and spleen from *Hdc*-CreERTM mice at D3, D7, D14 after limb ischemia. n=3 biological replicates. (d) Representative image of FASC analysis of EGFP$^+$ and CD11b$^+$ cell percentage in EGFP$^+$ cells of peripheral blood and bone marrow from ischemic *Hdc*-CreERTM mice, non-ishchemic *Hdc*-CreERTM mice and WT mice at D3, D7, D14 after limb ischemia. (e) FACS analysis of relative proportion of the CD11b$^+$ cells and CD11b$^+$Ly6C$^+$ monocytes in GFP$^+$ cells in peripheral blood of *Hdc*-CreERTM mice at D3, D7, D14 after limb ischemia. n = 3 mice per group. For all experiments, error bars represent mean±SD. $*p < 0.05$, $**p < 0.01$, $***p < 0.001$, $****p < 0.0001$.

Figure S3

a

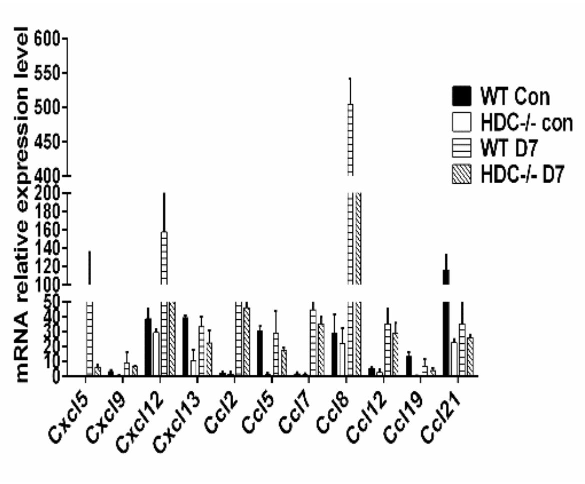

b

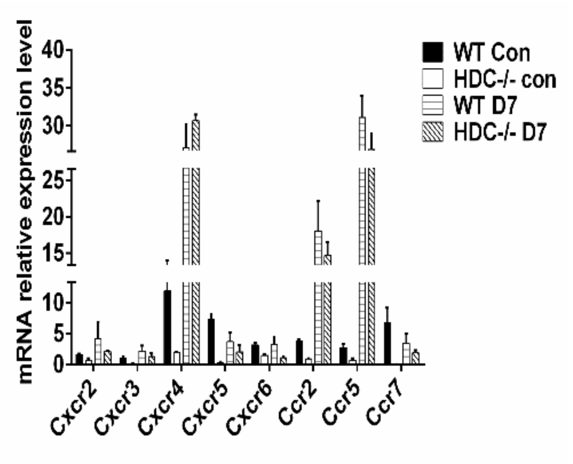

Figure S3 (a-b) Chemokines and chemokine receptors mRNA expression were tested in the ischemic muscle from Hdc-/- and WT mice at D7 after ischemia by RNA-Seq analysis. n = 3 biological replicates.

Figure S4

a

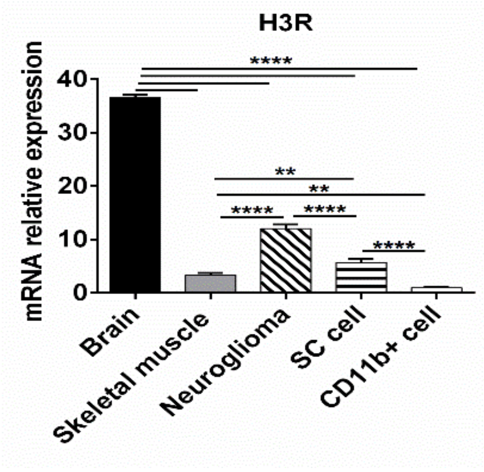

Figure S4 (a) mRNA expression level of histamine H3 receptor in the skeletal muscle, immune cells, C2C12, neuroglioma cell line (U87) and brain tissue.

PCR primers

Gene	Forward primer	Reverse primer
Gapdh	CCACTCACGGCAAATTCAAC	GTAGACTCCACGACATACTCAG
Il-1β	CAAGCAACGACAAAATACCTGTG	AGACAAACCGTTTTTCCATCTTCT
Il-6	CCGGAGAGGAGACTTCACAGAG	CTGCAAGTGCATCATCGTTGTT
Tnf-α	TGGCCCAGACCCTCACACTCAG	ACCCATCGGCTGGCACCACT
Il-10	CCATGGCCCAGAAATCAAGG	TCTTCACCTGCTCCACTGCC
Tgf-β	ACCGGAGAGCCCTGGATACCA	TATAGGGGCAGGGTCCCAGACA
Igf-1	CGTCAGAACCAGTCCCATCT	ACAAACAGCCCAGAGCAAAC
Pi3k	CTACCCATCACAGCACATGG	GGGAGCAGTTGGTGACATCT
Akt	AGCCCGAAGTCCGTTATCTT	GATTGTGTCTGCCCTGGACT
Pkd1	GCCAAGGCCTTAAATGTGAA	GGAGCTTGTCGAGCTGAATC
P70s6k	CCCACTGCTTTGAGCTACT	CTGAGGCACTCCAGGATG
